# PKR and TLR3 trigger distinct signals that coordinate the induction of antiviral apoptosis

**DOI:** 10.1038/s41419-022-05101-3

**Published:** 2022-08-15

**Authors:** Wenjie Zuo, Mai Wakimoto, Noriyasu Kozaiwa, Yutaro Shirasaka, Seong-Wook Oh, Shiori Fujiwara, Hitoshi Miyachi, Amane Kogure, Hiroki Kato, Takashi Fujita

**Affiliations:** 1 Division of Integrated Life Science, Graduate School of Biostudies, Kyoto University, Sakyo-ku, Kyoto, 606-8507 Japan; 2 Laboratory of Regulatory Information, Institute for Frontier Life and Medical Science, Kyoto University, Sakyo-ku, Kyoto, 606-8507 Japan; 3 Institute for Virus Research, Kyoto University, Sakyo-ku, Kyoto, 606-8507 Japan; 4 Institute for Cardiovascular Immunology, University Hospital Bonn, Bonn, 53127 Germany

**Keywords:** Cell death and immune response, Signal transduction, Pattern recognition receptors, Oligo delivery, Imaging the immune system

## Abstract

RIG-I-like receptors (RLRs), protein kinase R (PKR), and endosomal Toll-like receptor 3 (TLR3) sense viral non-self RNA and are involved in cell fate determination. However, the mechanisms by which intracellular RNA induces apoptosis, particularly the role of each RNA sensor, remain unclear. We performed cytoplasmic injections of different types of RNA and elucidated the molecular mechanisms underlying viral dsRNA-induced apoptosis. The results obtained revealed that short 5′-triphosphate dsRNA, the sole ligand of RIG-I, induced slow apoptosis in a fraction of cells depending on IRF-3 transcriptional activity and IFN-I production. However, intracellular long dsRNA was sensed by PKR and TLR3, which activate distinct signals, and synergistically induced rapid apoptosis. PKR essentially induced translational arrest, resulting in reduced levels of cellular FLICE-like inhibitory protein and functioned in the TLR3/TRIF-dependent activation of caspase 8. The present results demonstrated that PKR and TLR3 were both essential for inducing the viral RNA-mediated apoptosis of infected cells and the arrest of viral production.

## Introduction

During viral infection, viruses produce RNA species that are distinct from those of the host. Viral double-stranded RNA (dsRNA) is representative of non-self RNA and is recognized by sensor molecules belonging to the family of pattern recognition receptors (PRRs). dsRNA in the cytosol or extracellular space is recognized by different PRRs, resulting in type I interferon (IFN-I) production, and in some cases, apoptosis, which are considered to serve as antiviral innate immune responses [1, 2]. Retinoic acid-inducible gene I (RIG-I)-like receptors (RLRs), including RIG-I, melanoma differentiation-associated protein 5 (MDA5), and laboratory of genetics and physiology (LGP2), sense cytosolic dsRNA [3–7]. Long and short dsRNA are sensed by MDA5 and RIG-I, respectively [8]. Some viruses produce 5’-triphosphate-containing dsRNA (5’-ppp-RNA), which is preferentially sensed by RIG-I in the cytoplasm; host RNA species are processed to avoid the 5’-ppp structure [9, 10]. Upon sensing dsRNA, RIG-I and MDA5 alter their conformation and relay signals to the mitochondrial adaptor, interferon-β promoter stimulator 1 (IPS-1, also known as MAVS/VISA/Cardif). IPS-1 activates TANK-binding kinase 1 (TBK1), which catalyzes the activation of interferon regulatory factor 3 (IRF-3). IRF-3 is responsible for the production of IFN-I and antiviral proteins that limit viral replication [11–13]. Another PRR, Toll-like receptor 3 (TLR3), also senses dsRNA in the endosome and extracellular space, and activates IRF-3 through the adaptor, TIR-domain-containing adapter-inducing interferon-β (TRIF), and TBK1 to induce the common antiviral program, including IFN-I production [14–16].

dsRNA is also sensed by protein kinase activated by RNA (PKR); however, PKR has distinct functions from RLRs or TLR3. Upon activation by cytosolic dsRNA, PKR induces the phosphorylation of eukaryotic translation initiation factor 2 (eIF2a), which results in translational arrest, leading to the suppression of viral replication and cellular stress responses, such as apoptosis [17–20]. This process ensures viral eradication and the suppression of excessive inflammation. Although PKR activation per se does not induce the production of IFN-I, it facilitates viral RNA sensing by RLRs by inducing the formation of stress granules (SGs) [21].

The artificial dsRNA mimic, poly I:C, has been used to stimulate cells for IFN-I production due to its potent activity and common availability. When poly I:C is added to culture medium, it is taken up by cells into endosomes and activates TLR3. However, 5’-ppp-RNA or poly I:C has to be transfected into cells to activate RLRs and PKR. These stimuli commonly induce the production of IFN-I; however, the production of IFN-I and induction of apoptosis do not necessarily coincide, suggesting that these phenomena are regulated by distinct mechanisms. The intracellular delivery of poly I:C was previously reported to strongly induce apoptosis by undefined mechanisms in tumor cells [22–25]. However, the role of 5’-ppp-RNA in apoptosis remains unclear. Regarding the involvement of IRF-3 in the induction of apoptosis, its transcriptional activity-dependent and -independent mechanisms have been reported in viral infection [26, 27]. PKR has also been shown to regulate apoptosis through intrinsic and extrinsic pathways by signaling the BCL-2 family proteins, Bak and Bax, or caspase 8 [19, 28–32]. TLR3-TRIF signaling promotes the assembly of the death-inducing signaling complex (DISC), in which the oligomerization of caspase 8 induces apoptosis [33, 34]. Viral infection leads to the simultaneous activation of multiple receptors; however, the precise function of each receptor and component in dsRNA-induced apoptosis has not yet been established.

Therefore, this study investigated the mechanisms underlying dsRNA-induced cell death, particularly the involvement of RLRs, TLR3, and PKR. We utilized microinjections to introduce RNA/protein into the cytoplasm and observed sequential events in a single cell. Microinjections induce rapid and undisputed cellular responses in cells with limited transfection efficiency. We generated knockout (KO) cell lines for critical signaling components to elucidate their precise roles in programmed cell death. We demonstrated that the collaboration of PKR and TLR3 was essential in cytosolic dsRNA-induced apoptosis. The present results also suggest that viral RNA-induced cell death is a host mechanism that limits viral replication together with the direct effects of the antiviral mechanism induced by IFN-I.

## Results

### Induction of the nuclear translocation of IRF-3 followed by *IFNB* gene expression after the cytoplasmic injection of RNA/protein

**Fig. 1:**
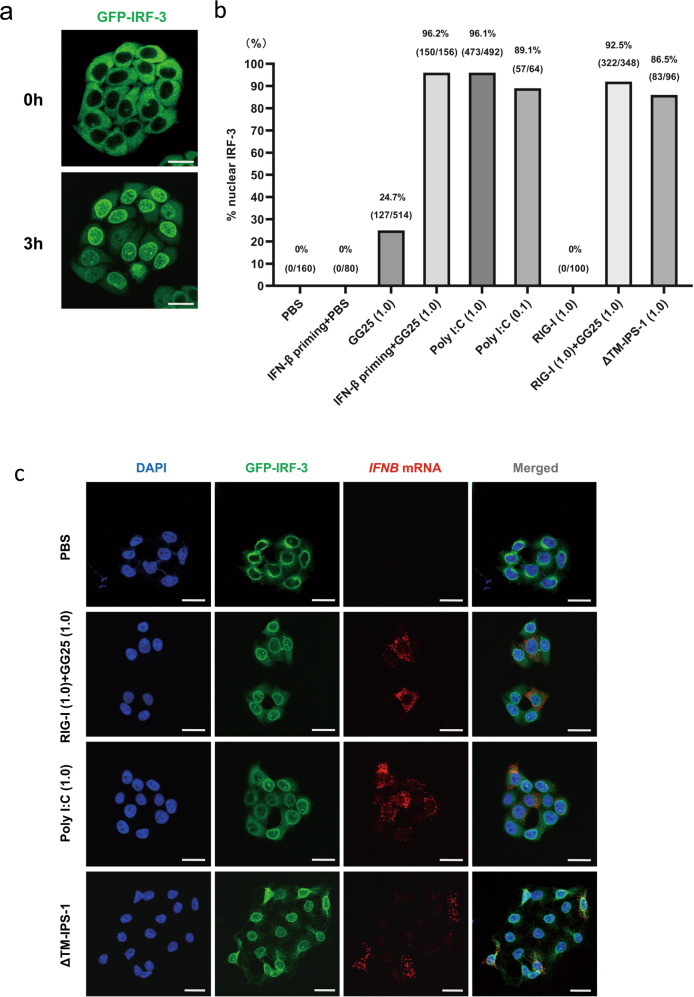
The cytoplasmic RNA/protein injection induces prompt and potent cellular responses. **a** GFP-IRF-3 HeLa cells were injected with poly I:C (1 μg/μl) and observed live for the localization of IRF-3 at the indicated times after the injection. The injection was performed for a colony of cells, each cell was followed for the nuclear translocation of IRF-3, and the % of nuclear IRF-3 was calculated (number of cells with nuclear IRF-3/number of injected cells). **b** GFP-IRF-3 HeLa cells were injected with the indicated RNA/protein. GG25: 5’-ppp-RNA; poly I:C: long poly I:C; RIG-I: recombinant RIG-I protein; ΔTM-IPS-1: recombinant IPS-1 protein devoid of the transmembrane domain. The amount of injected RNA or protein (μg/μl) is indicated at the bottom of the graph. IFN-β priming indicates the pretreatment of cells with IFN-β (1000 U/ml for 12 h) prior to the injection. At 3 h, injected cells were observed live for the localization of IRF-3, and % nuclear IRF-3 was calculated as in (**a**) and indicated at the top of each bar. **c** GFP-IRF-3 HeLa cells were injected with the indicated RNA/protein. Cells were fixed after 3 h and observed for IRF-3 (green) or the expression of *IFNB* mRNA (FISH, red). Scale bar = 25 µm.

The transfection of in vitro transcribed short 5’-ppp-RNA (GG25) and the dsRNA mimic (poly I:C) strongly induced IRF-3 activation and subsequent *IFNB* gene expression in HeLa cells [35]. We generated HeLa cells lacking endogenous IRF-3 and expressing GFP-tagged IRF-3 to monitor the localization of IRF-3 in real-time (“Materials and methods”). To analyze the outcomes of stimuli by these RNAs in individual cells, we stimulated cells by microinjections and each cell was followed by the live cell imaging of GFP (Supplementary Fig. S1a). A typical result of IRF-3 translocation induced by the poly I:C injection is shown in Fig. 1a. GFP-IRF-3 HeLa cells were injected with stimulant RNA or proteins and monitored for the nuclear translocation of IRF-3 (Fig. 1b). The injection of PBS did not affect the cytoplasmic localization of IRF-3. Poly I:C strongly induced the nuclear translocation of IRF-3 (89–96%). The GG25 injection induced nuclear IRF-3 in a fraction of cells (25%); however, IFN-β priming prior to the GG25 injection markedly increased the efficiency of IRF-3 activation (96%). Similarly, the injection of recombinant RIG-I protein, which did not activate IRF-3, efficiently promoted IRF-3 activation by the GG25 co-injection (93%). These results are consistent with RIG-I being interferon-inducible and increases in RIG-I levels promoting the sensing of GG25 to trigger antiviral signaling. In addition, the injection of ΔTM-IPS-1 protein efficiently activated IRF-3, which is consistent with previous findings showing that an in vitro incubation of ΔTM-IPS-1 with a cell extract activated the dimerization of IRF-3 [36]. *IFNB* gene activation in cells with nuclear IRF-3 was confirmed by fluorescence in situ hybridization (FISH) analysis (Fig. 1c). The injection of GG25 triggered *IFNB* gene expression in some cells expressing nuclear IRF-3 (Fig. S1b); however, overall efficiency was enhanced by the co-injection of RIG-I + GG25 (Fig. S1c). These results indicated that the delivery of RNA/protein into the cell cytoplasm by microinjections triggered rapid and potent cellular responses, and, thus, is suitable for single-cell analysis.

### Induction of cell death by the cytoplasmic injection of RNA/protein

**Fig. 2:**
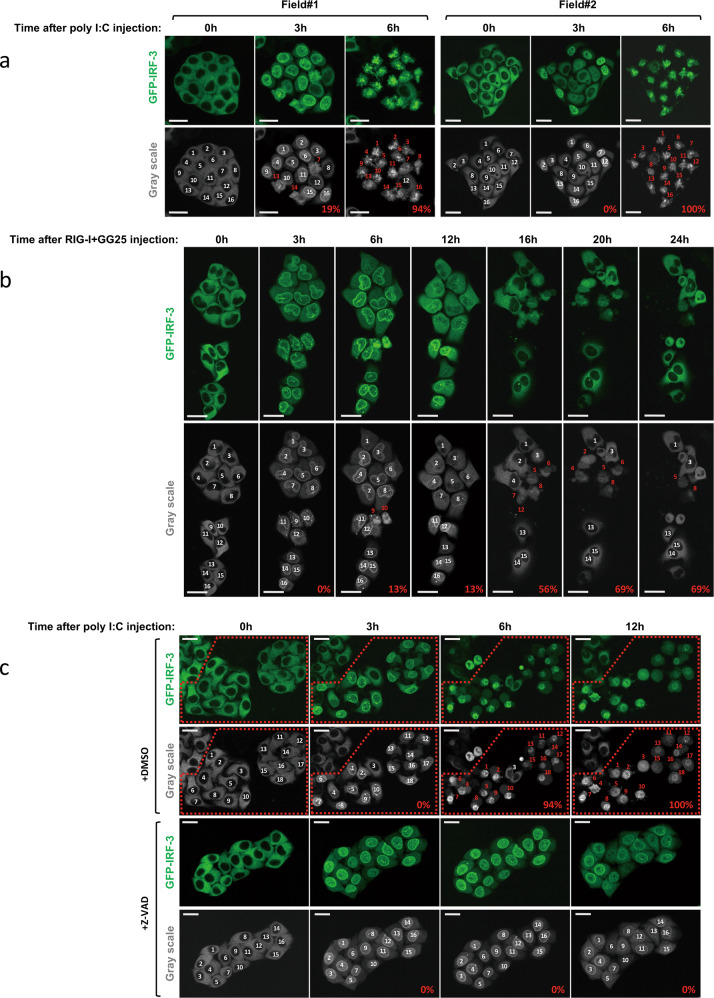
Induction of the nuclear translocation of IRF-3 and subsequent cell death by the cytoplasmic injection of RNA/protein. **a** GFP-IRF-3 HeLa cells were injected with poly I:C (1 μg/μl) and observed live for the localization of IRF-3 at the indicated times after the injection. Injected cells were numbered (white) and followed; cells with red numbers exhibited morphological cell death; % of dead cells was calculated (as depicted in grayscale images). **b** GFP-IRF-3 HeLa cells were injected with RIG-I and GG25 (1 μg/μl each) and observed as in (**a**). **c** GFP-IRF-3 HeLa cells were injected with poly I:C (1 μg/μl) in the absence (DMSO) or presence of Z-VAD (20 µΜ) and observed as in (**a**). Z-VAD was added 3 h prior to the injection and kept in the culture medium. Injected cells are indicated in the red dotted box. Scale bar = 25 µm.

We further investigated cell fate after the activation of IRF-3 by an RNA injection. When poly I:C was injected into GFP-IRF-3 HeLa cells, cells exhibited efficient IRF-3 nuclear translocation at 3 h and these cells underwent robust cell death at 6 h (Fig. 2a). The cell death was not due to the mechanical stress of the injection because it occurred 3–6 h after the injection and the PBS injection did not induce cell death (Supplementary Fig. S2a). Moreover, mechanical damage resulted in the release of cytoplasmic contents, including GFP-IRF-3 (Supplementary Fig. S2a, b). Cells were injected with RIG-I + GG25 to analyze cell death by RIG-I signaling (Fig. 1b). Injected cells exhibited the nuclear translocation of IRF-3 and cell death; however, in contrast to the poly I:C injection, its kinetics were slow, and with lower efficiency (Fig. 2b). Furthermore, surviving cells that once exhibited nuclear IRF-3 at 3–12 h showed its re-location back to the cytoplasm at 16–24 h (Fig. 2b). To examine the type of cell death observed, we treated cells with Z-VAD, a pan-caspase inhibitor (Fig. 2c). Cells pre-treated with Z-VAD and then injected with poly I:C exhibited the efficient nuclear translocation of IRF-3; however, cell death was not induced as late as 12 h after the injection. This result suggested that cell death observed after the cytoplasmic poly I:C injection was apoptosis. To establish whether the cytoplasmic introduction of RNA by transfection resulted in a similar outcome, we transfected these RNAs into GFP-IRF-3 HeLa cells (Supplementary Fig. S3a). The transfection of poly I:C induced massive apoptosis within 24 h, whereas that of GG25 alone only induced limited cell death. IFN-β priming, which induced the expression of RIG-I, enhanced cell death upon the transfection of GG25 (Supplementary Fig. S3b). Similarly, priming enhanced poly I:C transfection-induced cell death (Supplementary Fig. S3c). However, the kinetics of cell death induced by GG25 and poly I:C were slow and fast, respectively, irrespective of priming. These results suggest that these RNAs induce cell death through distinct mechanisms. In contrast to injections, exogenous treatment of poly I:C induced nuclear IRF-3, but no significant cell death in HeLa cells (see below). Therefore, the apoptosis observed was induced by a series of physiological signals in response to different types of cytosolic RNA.

### Diverse mechanisms underlying cell death induced by GG25 and poly I:C

**Fig. 3:**
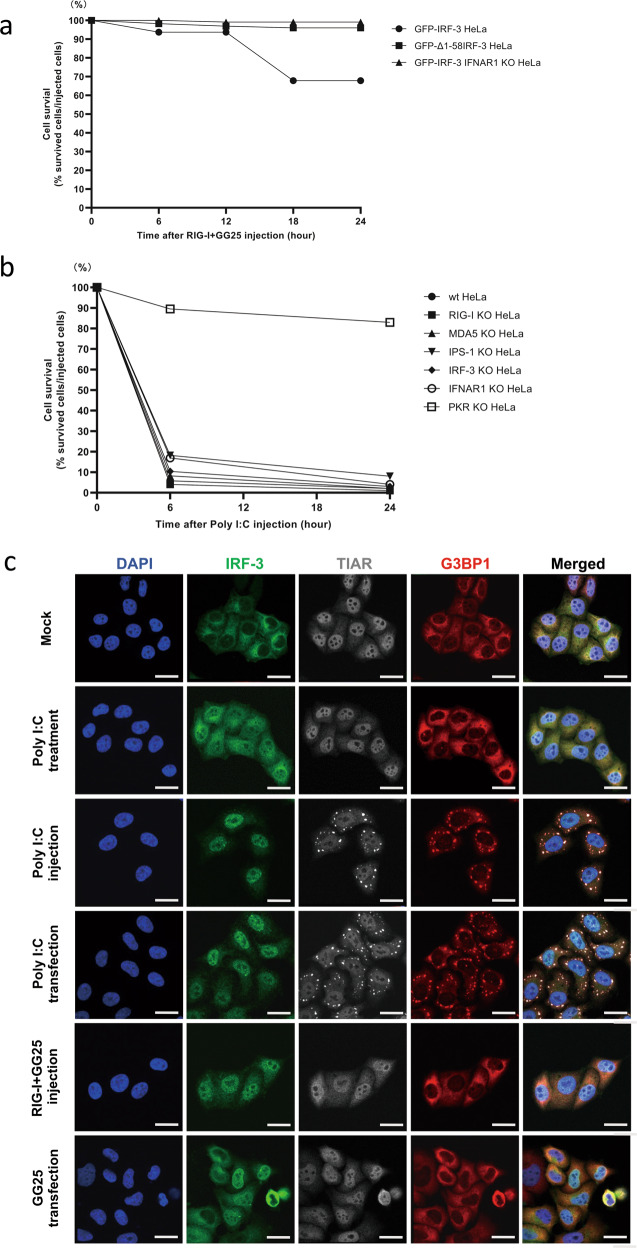
Diverse mechanisms of cell death induced by GG25 and poly I:C. **a** GFP-IRF-3 HeLa, GFP-Δ1-58IRF-3 HeLa, and GFP-IRF-3 IFNAR1 KO HeLa cells were injected with RIG-I and GG25 (1 μg/μl each), observed live for cell death, and quantified for % cell survival at the indicated time points. **b** HeLa cells and indicated KO HeLa cells were injected with poly I:C (1 μg/μl), observed for cell death, and % cell survival was quantified. **c** GFP-IRF-3 HeLa cells were mock treated, treated with poly I:C (5 μg/ml in culture medium for 3 h), injected with poly I:C (1 μg/μl for 3 h), transfected with poly I:C (0.5 μg/ml in culture medium with lipofectamine for 3 h), injected with RIG-I and GG25 (1 μg/μl each for 3 h), or transfected with GG25 (4 μg/ml in culture medium with lipofectamine for 3 h). Cells were fixed and stained for TIAR and G3BP1 with respective antibodies for microscopy. Scale bar = 25 µm.

To investigate the mechanistic differences in cell death induced by poly I:C and RIG-I + GG25, we examined the role of transcriptional activation by IRF-3 in cell death. We expressed GFP-Δ1-58IRF-3 in IRF-3 KO HeLa cells (GFP-Δ1-58IRF-3 HeLa) (Supplementary Fig. S4a). Δ1-58IRF-3 did not exhibit transcriptional activity (Supplementary Fig. S4a) and has been shown to function as a dominant inhibitor [37]. GFP-IRF-3 HeLa and GFP-Δ1-58IRF-3 HeLa were injected with RIG-I + GG25 and cell death was then examined (Fig. 3a). Cell death was limited in GFP-IRF-3 HeLa cells and absent in GFP-Δ1-58IRF-3 cells. Furthermore, cell death induced by RIG-I + GG25 was inhibited by deleting IFNAR1 (GFP-IRF-3 IFNAR1 KO HeLa). Similarly, the transfection of GG25 induced the prominent cleavage of PARP in GFP-IRF-3 HeLa cells; however, cleavage was markedly attenuated in GFP-Δ1-58IRF-3 and GFP-IRF-3 IFNAR1 KO HeLa cells (Supplementary Fig. S4b). This result suggested that cell death mediated by RIG-I was largely dependent on the transcriptional activity of IRF-3 and the effects of secreted type I IFN, and is consistent with the slow time course of cell death after the RIG-I + GG25 injection.

In contrast, when GFP-Δ1-58IRF-3 and GFP-IRF-3 IFNAR1 KO HeLa cells were injected with poly I:C, efficient cell death was observed, suggesting that cell death induced by poly I:C was independent of the activation of IRF-3 target genes and type I IFN (Supplementary Fig. S4c). To further investigate the involvement of signaling components in cell death, we used HeLa cells lacking RIG-I, MDA5, IPS-1, IRF-3, IFNAR1, or PKR (Fig. 3b). These cells, except for PKR KO, exhibited efficient cell death upon the poly I:C injection, suggesting that cell death was dependent on PKR, but independent of RLR or type I IFN signaling. Similar results were obtained with the transfection of poly I:C (Supplementary Fig. S4d). We confirmed that cell death was not due to the synthetic nature of poly I:C by injecting natural rice *Endornavirus* dsRNA (rb-dsRNA) extracted from rice bran (“Materials and methods” and Supplementary Fig. S4e) [38]. We then investigated whether SG, the formation of which is induced by activated PKR, was detectable after the introduction of different RNA by different methods (Fig. 3c). The efficient nuclear translocation of IRF-3 was induced in HeLa cells treated with poly I:C without a transfection reagent. However, SG detected as cytoplasmic granules containing TIAR and G3BP1 were not induced. In contrast, the injection or transfection of poly I:C induced both nuclear translocation of IRF-3 and SG. These results further supported PKR being essential in cell death induced by the poly I:C injection or transfection and are consistent with the poly I:C treatment inducing neither cell death nor SG. GG25 induced the nuclear translocation of IRF-3, but not SG, by injection or transfection, indicating that the mechanisms underlying cell death induced by GG25 and poly I:C were distinct. Since efficient and rapid cell death was induced, we thereafter focused on the mechanisms underlying cell death induced by poly I:C.

### Synergistic induction of cell death by PKR and the exogenous poly I:C treatment

**Fig. 4:**
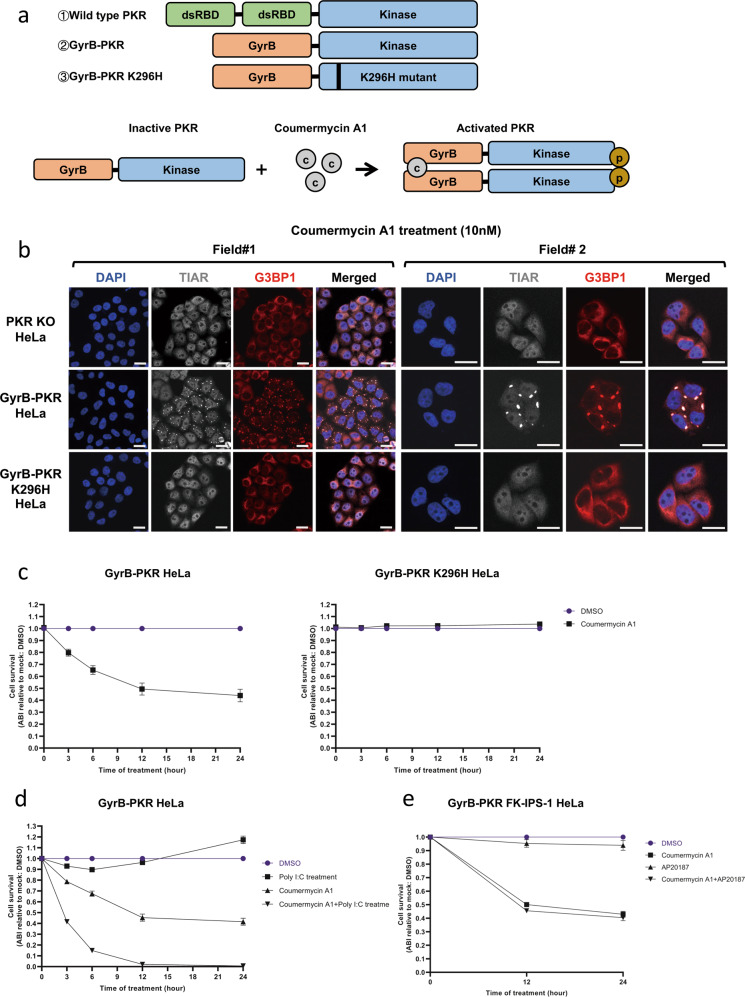
Synergistic induction of cell death by PKR and the exogenous poly I:C treatment. **a** Schematic representation of PKR and GyrB-PKR fusion proteins and the activation mechanism of GyrB-PKR fusion by coumermycin A1. **b** Induction of SG by the coumermycin A1 treatment. PKR KO HeLa, GyrB-PKR HeLa, and GyrB-PKR K296H HeLa cells were treated with coumermycin A1 (10 nM for 3 h) and observed for the SG marker TIAR (gray) and G3BP1 (red), as in Fig. 3c. Scale bar = 25 µm. **c** GyrB-PKR HeLa and GyrB-PKR K296H HeLa cells were mock treated (DMSO) or treated with coumermycin A1 (10 nM) for the indicated times and examined for cell survival. **d** GyrB-PKR HeLa cells were treated with the indicated chemicals and examined for cell survival at each time point. **e** GyrB-PKR FK-IPS-1 HeLa cells were treated with the indicated chemicals and examined for cell survival at each time point. Cell survival in (**c**–**e**) was examined by the Amido black assay (“Materials and methods”) and presented as Amido black intensity (ABI) relative to the mock. Data are represented as the means ± SEM of three independent experiments.

The strong apoptosis induced by cytosolic dsRNA in a PKR-dependent and RLR-independent manner was of interest. To further clarify the role of PKR, we utilized a system in which PKR activity is inducible by the small molecule, coumermycin A1 [39, 40]. We generated PKR KO HeLa cells expressing the fusion protein GyrB-PKR or GyrB-PKR K296H (kinase-dead mutant) to examine the effects of the sole activation of PKR without using dsRNA (Fig. 4a and Supplementary Fig. S5a). In GyrB-PKR, the dsRNA-binding domain of PKR was replaced by the bacterial Gyrase B subunit (Fig. 4a). When coumermycin A1 was added to the cell culture, the GyrB subunit dimerized, and PKR was activated (Fig. 4a). The activation of PKR by coumermycin A1 was confirmed by the induction of SG in GyrB-PKR HeLa, but not in PKR KO HeLa or GyrB-PKR K296H HeLa cells (Fig. 4b). A quantitative analysis of cell survival (Fig. 4c) demonstrated that the activation of PKR alone induced significant cell death; however, it was less prominent (45%) than that induced by the poly I:C injection (>95%) (Fig. 3b). A higher concentration of coumermycin A1 (100 nM) did not promote further cell death (Supplementary Fig. S5b). We then examined the effects of the poly I:C treatment, which activates endosomal TLR3, but not PKR (Fig. 3c). As predicted, the poly I:C treatment did not induce major cell death over time, while the coumermycin A1 treatment resulted in partial cell death (Fig. 4d). The combined treatment of cells with poly I:C and coumermycin A1 induced nearly complete cell death within 12 h (Fig. 4d). We also investigated whether the combined activation of the PKR and RLR pathways exerted synergistic effects on the induction of cell death. The FKBP fusion system has been used to selectively activate RLR signaling (Supplementary Fig. S5c) [41]. We expressed FK-IPS-1 in GyrB-PKR HeLa cells (Supplementary Fig. S5d) and treated them with coumermycin A1, AP20187, or their combination (Fig. 4e). The activation of RLR signaling by AP20187 was confirmed by the efficient nuclear translocation of IRF-3 in AP20187-treated cells and the activation of PKR was confirmed by the induction of SG (Supplementary Fig. S5d). The lack of significant synergy by the activation of PKR and IPS-1 was confirmed (Fig. 4e).

### Involvement of TLR3 signaling in cell death induced by cytoplasmic poly I:C

**Fig. 5:**
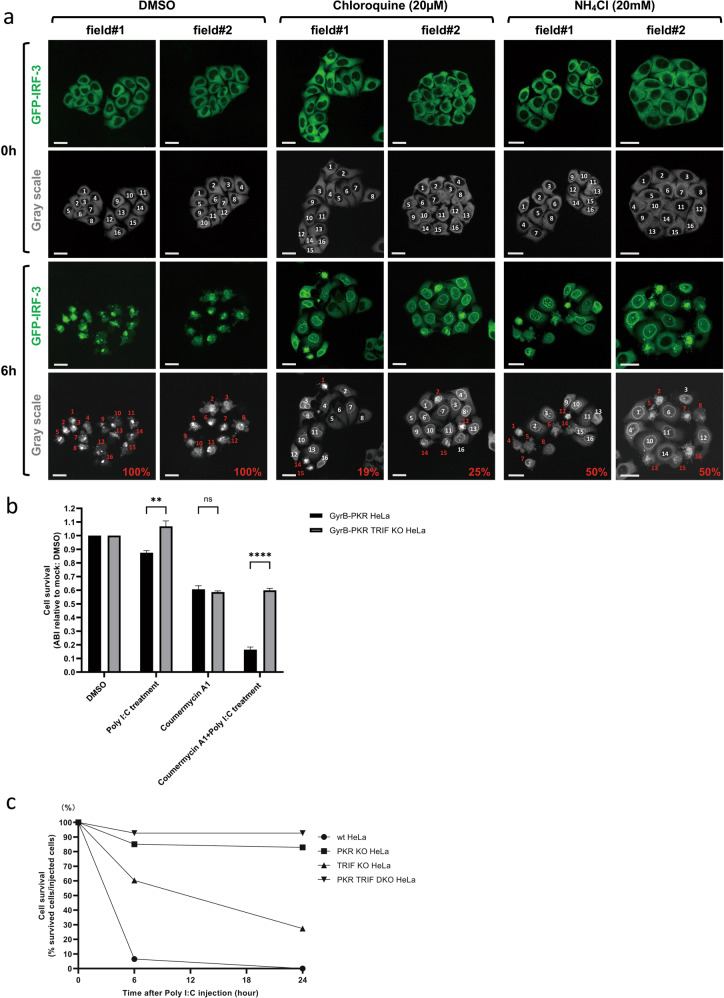
Involvement of TLR3 signaling in cell death induced by cytoplasmic poly I:C. **a** GFP-IRF-3 HeLa cells were treated with DMSO, chloroquine (20 μM for 6 h), or NH_4_Cl (20 mM for 6 h) and then injected with poly I:C (1 μg/μl). GFP images of live cells were taken 0 and 6 h after the poly I:C injection. In each field, cells were numbered, assessed as dead (red) or alive (white), and % cell death was calculated. Scale bar = 25 µm. **b** GyrB-PKR HeLa and GyrB-PKR TRIF KO HeLa cells were treated with the indicated chemicals for 6 h and examined for cell survival. The means ± SEM of three independent experiments are shown; data were analyzed by a two-way ANOVA followed by Tukey’s multiple comparisons test; **P < 0.01, ****P < 0.0001; ns not significant. **c** Wild-type, PKR KO, TRIF KO, and PKR TRIF DKO HeLa cells were injected with poly I:C (1 μg/μl), observed live for cell death, and % cell survival at the indicated time points was quantified.

Since the activation of PKR and the poly I:C treatment exerted strong synergistic effects on the induction of cell death, we hypothesized that PKR and TLR3 signaling cooperate to promote cell death practically under the poly I:C injection. Since the injection did not result in sufficient extracellular leakage of poly I:C to trigger the translocation of IRF-3 in surrounding un-injected cells, we assumed that cytosolically injected poly I:C was also captured by endosomal TLR3. We used chloroquine and NH_4_Cl to inhibit endosomal acidification, which is essential for the activation of TLR3 by dsRNA [42, 43]. These chemicals attenuated cell death induced by the poly I:C injection (Fig. 5a). We then generated TRIF KO cells from GyrB-PKR HeLa cells (GyrB-PKR TRIF KO HeLa cells expressing GyrB-PKR lacking *PKR* and *TRIF*). GyrB-PKR and GyrB-PKR TRIF KO HeLa cells were exogenously stimulated with poly I:C, coumermycin A1, or both (Fig. 5b). Synergistic cell death in GyrB-PKR HeLa cells induced by the poly I:C and coumermycin A1 treatment was not observed in GyrB-PKR TRIF KO HeLa cells, suggesting the involvement of TRIF in cell death. HeLa cells and mutant cells lacking PKR, TRIF, or both PKR and TRIF (double KO (DKO)) were generated (Supplementary Fig. S6a) and subjected to stimulation by the poly I:C injection (Fig. 5c). Prominent cell death in wild-type HeLa cells was partially blocked by the deletion of TRIF, and full inhibition was noted in DKO cells. A similar result was obtained by the transfection of poly I:C in these cells (Supplementary Figs. S6b and S7a). Note that TRIF KO did not affect the activation of PKR based on the formation of SG (Supplementary Fig. S6c). This is consistent with our hypothesis that cytoplasmic poly I:C activates PKR and endosomal TLR3/TRIF signaling to strongly induce cell death.

### PKR activation resulted in the downregulation of cFLIP and promoted the endosomal poly I:C-induced activation of caspases 8 and 9

**Fig. 6:**
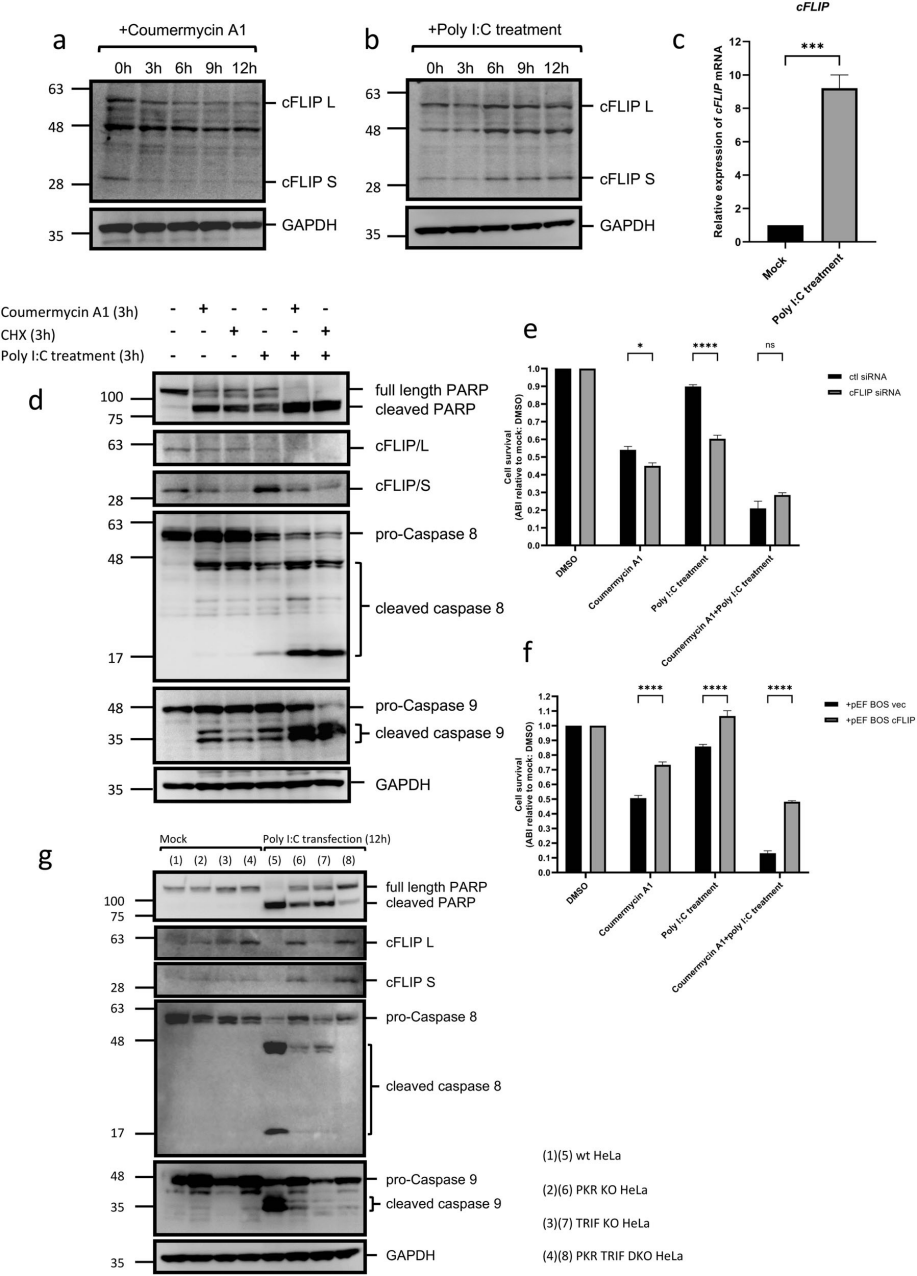
PKR activation resulted in the downregulation of cFLIP and promoted the endosomal poly I:C-induced activation of caspases 8 and 9. **a** GyrB-PKR HeLa cells were treated with coumermycin A1 for the indicated times and examined for the levels of cFLIP L, cFLIP S, and GAPDH by immunoblotting. **b** GyrB-PKR HeLa cells were treated with poly I:C (5 μg/ml) and examined for the levels of cFLIP L, cFLIP S, and GAPDH by immunoblotting. **c** GyrB-PKR HeLa cells were left untreated or treated with poly I:C (5 µg/ml, 6 h). Cells were harvested and the relative expression of the *cFLIP* gene was examined by RT-qPCR. The means + SEM of three independent experiments are shown; data were analyzed by an unpaired *t* test; ***P < 0.001. **d** GyrB-PKR HeLa cells were treated with the indicated chemicals (10 nM coumermycin A1; 10 µg/ml CHX; 5 μg/ml poly I:C) for 3 h and examined for the indicated proteins by immunoblotting. **e** GyrB-PKR HeLa cells were transfected with control (ctl) or specific siRNA for cFLIP for 48 h, treated with the indicated chemicals as in (**d**), and examined for cell survival. **f** GyrB-PKR HeLa cells were transfected with the control (pEF-BOS vec) or expression vector for cFLIP (pEF-BOS cFLIP) for 48 h, treated with the indicated chemicals as in (**d**), and examined for cell survival. **g** Wild-type, PKR KO, TRIF KO, and PKR TRIF DKO HeLa cells were transfected with poly I:C (0.5 µg/ml) for 12 h and examined for the indicated proteins by immunoblotting. Cell survival in (**e**, **f**) is presented as ABI relative to the mock, and the means ± SEM of three independent experiments are shown; data were analyzed by a two-way ANOVA followed by Tukey’s multiple comparisons test; *P < 0.05, ****P < 0.0001; ns not significant. Refer to full-length western blot images in Supplemental Materials.

Previous studies reported that the activation of TLR3 directly induced the TRIF-FADD-caspase 8 cascade of apoptosis in certain cell types [33, 44, 45]. However, FADD-caspase 8 signaling complex (DISC) was previously shown to be inhibited by the expression of cellular FADD-like inhibitory protein (cFLIP) in cancer cells, a protein that is sensitive to cycloheximide (CHX) [46–48]. The activation of PKR is known to induce the formation of SG, which recruit the translational machinery to halt protein synthesis [20]. We hypothesized that the activation of PKR downregulates cFLIP to promote cell death. To confirm this, we activated PKR by coumermycin A1 and examined cFLIP levels (Fig. 6a). As expected, cFLIP levels markedly decreased after the activation of PKR, particularly those of the cFLIP S isoform. The effects of cellular stimulation by endosomal poly I:C on cFLIP levels were then examined (Fig. 6b). cFLIP L levels did not markedly decrease during the poly I:C treatment for 12 h. In contrast, cFLIP S levels increased after the poly I:C treatment and the induction of *cFLIP* mRNA was also detected upon the poly I:C treatment (Fig. 6c). When cells were treated with coumermycin A1, CHX, or poly I:C, the apoptosis markers, PARP, caspase 8, and caspase 9 were partially cleaved (Fig. 6d). However, when the poly I:C treatment was combined with coumermycin A1 or CHX, cleavage was markedly accelerated. The induction of cFLIP/S by the poly I:C treatment was canceled by the co-treatment with coumermycin A1 or CHX. These results suggest that cFLIP levels play a critical role in cell death.

To investigate the role of cFLIP in cell death mediated by PKR and TRIF, we manipulated cFLIP levels by siRNA and overexpression. The activation of PKR induced cell death, which was weakly promoted by the knockdown of cFLIP (Fig. 6e). The stimulation of TLR3 alone did not strongly induce cell death; however, the same stimulus promoted cell death in cells with the knockdown of cFLIP. The combined activation of PKR and TLR3 strongly induced cell death, whereas the knockdown of cFLIP did not further promote cell death. The effects of the overexpression of cFLIP were then examined (Fig. 6f). The overexpression of cFLIP significantly protected cells from cell death induced by different stimuli. Cell death induced by the activation of PKR was partially restored by the overexpression of cFLIP, but not to the level of non-stimulated cells. The strong induction of cell death by the activation of PKR and TLR3 was significantly inhibited by the overexpression of cFLIP, albeit incompletely. This result indicated that the activation of PKR promoted cell death, at least in part, by downregulating cFLIP. PKR has also been suggested to promote cell death independent of cFLIP and TLR3. The roles of PKR and TLR3/TRIF in the regulation of cFLIP and the further synergistic activation of apoptosis were confirmed by the transfection of poly I:C in wild-type and KO HeLa cells (Fig. 6g). The downregulation of cFLIP was not observed in cells lacking PKR, and the production of the apoptotic markers, cleaved PARP, and caspases 8 and 9 was partially reduced in single KO HeLa cells and abrogated in DKO HeLa cells. In comparison with its injection, the transfection of poly I:C in wild-type and KO HeLa cells delayed the onset of apoptosis (Supplementary Fig. S7a). In cells transfected with poly I:C, the levels of apoptosis markers (Fig. 6g) were consistent with the degree of cell death observed (Supplementary Figs. S7a and 12h).

### Roles of PKR and TLR3/TRIF in virus-induced cell death and viral yield

**Fig. 7:**
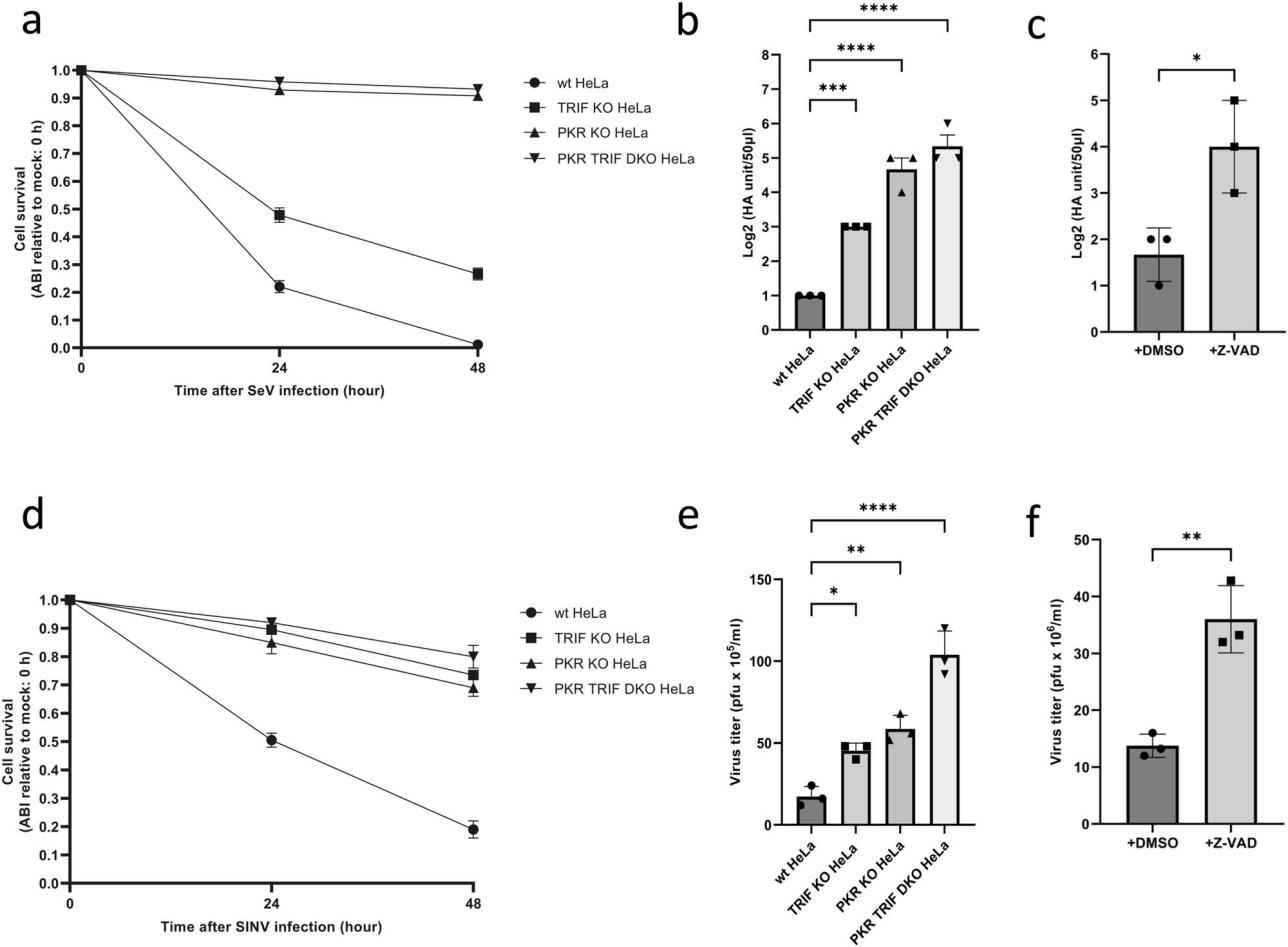
Roles of PKR and TLR3/TRIF in virus-induced cell death and viral yield. **a** Wild-type, PKR KO, TRIF KO, and PKR TRIF DKO HeLa cells were mock treated or infected with Sendai virus (SeV) for 48 h, and quantified for cell survival. **b** HA yield in the culture supernatant of cells infected in (**a**) were examined. **c** Wild-type HeLa cells were infected with SeV with or without Z-VAD (50 μM) in culture medium for 72 h, and the culture supernatant was examined for HA yield. **d** The same set of cells in (**a**) were mock treated or infected with Sindbis virus (SINV) for 48 h and quantified for cell survival. **e** Viral titers in the culture supernatant of cells in (**d**) were examined by a plaque assay. **f** Wild-type HeLa cells were infected with SINV with or without Z-VAD (50 μM) in culture medium for 72 h, and viral titers in the culture supernatant were examined by the plaque assay. The means ± SEM of three independent experiments are shown; data in (**b**, **d**) were analyzed by a one-way ANOVA followed by Dunnett’s multiple comparisons test; data in (**c**, **f**) were analyzed by an unpaired *t* test; *P < 0.05, **P < 0.01, ***P < 0.001, ****P < 0.0001.

**Fig. 8:**
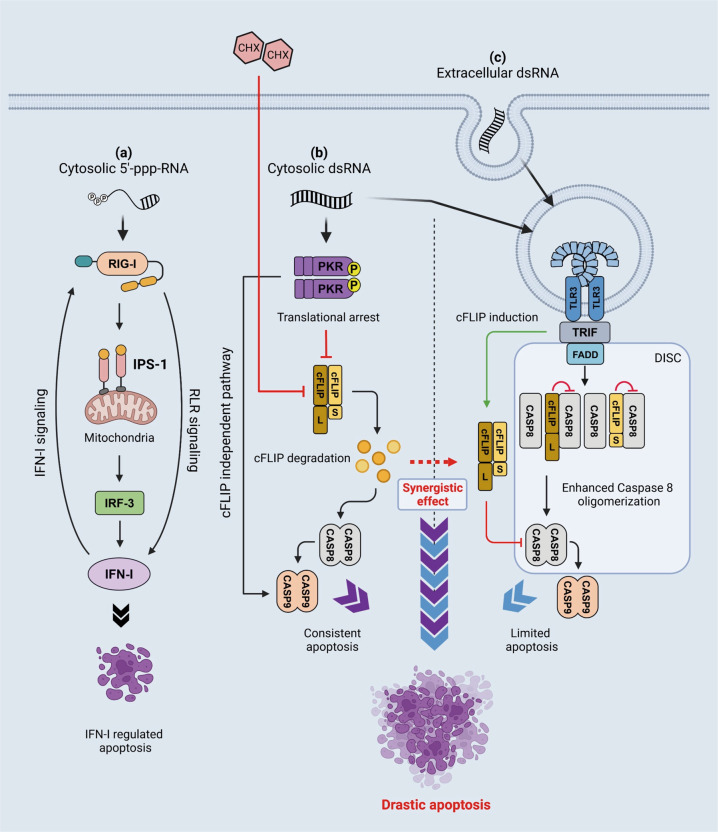
Schematic view of cell death signaling induced by 5’-ppp-RNA, cytosolic dsRNA, and extracellular dsRNA. **a** Cytosolic short 5’-ppp-dsRNA is sensed by RIG-I and activates signaling, leading to the induction of IFN-I and limited apoptosis with slow kinetics. This apoptosis is dependent on genes regulated by IRF-3 and IFN-I. **b** Cytosolic dsRNA introduced by the injection or transfection activates TLR3 through endosomal entrapment as above. In contrast to endosomal dsRNA, cytosolic dsRNA activates PKR, which results in the induction of SG and translational shutdown, leading to the downregulation of cFLIP and apoptosis through the activation of caspase 8. In addition, PKR was reported to promote the mitochondrial pathway in order to activate caspase 9; therefore, robust apoptosis was induced via caspases 8/9. **c** Extracellular dsRNA is incorporated by endocytosis and sensed by TLR3. TLR3 signals TRIF and activates signaling, leading to the induction of IFN-I and the expression of cFLIP in order to negatively regulate caspase 8. The TLR3 signaling complex also recruits FADD to activate DISC containing caspase 8; however, due to the effects of cFLIP, apoptosis is suppressed. When cells are treated with extracellular dsRNA and CHX, caspase 8 promotes apoptosis because of the downregulation of cFLIP.

dsRNA is absent in normal mammalian cells, but occasionally accumulates because of viral replication. Sendai virus (SeV), a negative-strand RNA virus, produces large amounts of dsRNA during infection. Therefore, we examined the roles of PKR and TLR3/TRIF in SeV-induced cell death (Fig. 7a) and viral yield (Fig. 7b). SeV-induced cell death was induced in wild-type HeLa cells, partially attenuated in TRIF KO HeLa cells, and strongly blocked in PKR KO and DKO HeLa cells. Concomitant with the inhibition of cell death, viral yield in the culture medium markedly increased in PKR KO and DKO HeLa cells (Fig. 7b). Importantly, the inhibition of cell death by the Z-VAD treatment increased viral yield in wild-type HeLa cells (Fig. 7c). This result confirmed that the induction of apoptosis directly contributed to viral inhibition. When Sindbis virus (SINV), a positive-strand RNA virus, was also examined, similar results were observed (Fig. 7d–f). Signaling by PKR and TRIF promoted virus-induced apoptosis and the suppression of viral replication. A schematic view of the viral dsRNA-induced antiviral apoptotic pathway is depicted in Fig. 8.

## Discussion

The accidental activation of programmed cell death is harmful for a host; therefore, it is tightly regulated by multiple mechanisms. The present study focused on the mechanisms underlying cell death induced by intracellular dsRNA.

The viral RNA sensor, RIG-I, senses relatively short dsRNA and induces signals to activate IFN-I. We used GG25, a 5’-ppp-dsRNA, to selectively activate RIG-I. The cytoplasmic injection of RIG-I + GG25 induced the robust nuclear translocation of IRF-3 and subsequent production of IFN-I (Fig. 1c). Among cells that exhibited nuclear IRF-3, no major cell death was detected within 12 h, and a fraction of cells (50–60%) underwent cell death 16 h after the injection (Fig. 2b). Our analyses revealed that the transcriptional activity of IRF-3 and secondary activation of genes by IFN-I were essential for the death signal induced by 5’-ppp-RNA (Fig. 3a). This result suggested that the activation of RIG-I essentially activated IFN-I, but did not trigger cell death by itself (Fig. 8a).

The cytoplasmic injection of poly I:C triggered the efficient nuclear translocation of IRF-3 (~100%) and strongly induced cell death within 6 h (Figs. 1b and 2a). Poly I:C has been used to stimulate cells for IFN-I via both transfection and exogenous treatments; however, the cell fates markedly differ (Supplementary Fig. S6b). The base composition of poly I:C is distinct from that of viral dsRNA. To exclude the possibility that cell death strongly induced by the poly I:C injection was due to its base sequence, we used natural rb-dsRNA to confirm the commonality of the cell death mechanism induced by dsRNA. The efficient rb-dsRNA-induced nuclear translocation of IRF-3 and rapid cell death were indistinguishable from those induced by poly I:C (Supplementary Fig. S4e). We identified PKR as an important regulator of cell death by examining a series of KO cell lines (Fig. 3b). Furthermore, we demonstrated that the artificial activation of PKR without using dsRNA was sufficient for the induction of cell death, and its kinase activity was essential (Fig. 4). These results are consistent with previous findings showing that PKR promoted a death signal through mitochondrial components [18, 32]. However, cell death promoted by PKR alone was less efficient (~50%, Fig. 4c) than the poly I:C injection (~100%, Fig. 3b). Therefore, we hypothesized that another signal induced by intracellular poly I:C cooperated with PKR.

We speculated that the third cell death pathway was from cytoplasmic dsRNA sensed by endosomal TLR3. Poly I:C added to the culture medium was taken up by endocytosis and activated TLR3. Activated TLR3 induced the nuclear translocation of IRF-3 (Fig. 3c), but not prominent cell death in HeLa cells over time (Fig. 4d). Rapid cell death was induced by the co-stimulation of TLR3 and PKR (Fig. 4d). The KO of the TLR3 adaptor protein TRIF attenuated apoptosis triggered by either the co-stimulation of TLR3 and PKR or the poly I:C injection (Fig. 5b, c), suggesting that injected poly I:C was also sensed by endosomal TLR3. A previous study demonstrated that activated PKR phosphorylated eIF2a and promoted SG, and the translation of mRNA was halted [18]. Therefore, we hypothesized that an inhibitory protein with a short half-life was involved in cell death induced by the poly I:C injection. We focused on cFLIP and analyzed the effects of the inhibition of translation by CHX and the activation of PKR on cFLIP levels. As expected, cFLIP levels decreased after these treatments (Fig. 6a, d). Moreover, the knockdown and overexpression of cFLIP promoted and attenuated cell death, respectively (Fig. 6e, f), suggesting that cFLIP is a negative regulator targeted by PKR.

Of note, reduced cFLIP L levels were also detected at an early stage of the activation of TLR3 (3 h) (Fig. 6b, d), and a transient reduction in cell viability was observed at 3–6 h (Fig. 4d). However, further cell death was not induced (Fig. 4d) by further increases in cFLIP S levels during the poly I:C treatment (Fig. 6b–d). These results suggested that the downregulation of cFLIP by TLR3 was limited and may be a result of the autocleavage of the cFLIP L/caspase 8 heterodimer during incomplete cell death signaling [49–51].

Caspases 8 and 9 are central executors of apoptosis. The activation of PKR (by coumermycin A1) and TLR3 (by poly I:C treatment) weakly promoted the cleavage of caspases 8 and 9, respectively, and combined stimuli resulted in the marked cleavage of these caspases (Fig. 6d). This is consistent with KO studies on PKR, TRIF, and their combination (Fig. 6g). Therefore, we concluded that cytosolic dsRNA triggered two pathways regulated by PKR and TLR3, both of which are required for the full execution of apoptosis. The inhibition of translation by CHX exerted similar effects to the activation of PKR on the regulators of cell death (Fig. 6d), suggesting that the main mechanism of action by PKR is translational arrest. Moreover, the overexpression of cFLIP did not fully rescue PKR activation-induced apoptosis (Fig. 6f), suggesting that a cFLIP-independent cell death signal is activated by PKR, possibly via a mitochondrial pathway. The regulation of cell death by two independent mechanisms may not be limited to that induced by dsRNA. TNF-α, which alone did not induce apoptosis, synergistically induced apoptosis when combined with translational arrest by CHX or the activation of PKR (Supplementary Fig. S7b). Since the phosphorylation of eIF2a is catalyzed by the stress-induced kinases, PKR, HRI, GCN2, and PERK, cellular stress may be conditional for the induction of programmed cell death. However, PKR-mediated signaling did not cooperate with that triggered by RIG-I for cell death (Fig. 4e), further confirming that the main function of RIG-I is to induce IFN-I and antiviral proteins.

We examined the impact of dsRNA-induced cell death on virus production. The results obtained revealed that TRIF and PKR were required for efficient cell death in infection by SeV and SINV, which are negative- and positive-strand RNA viruses (Fig. 7a, d). Importantly, the viral titer was increased by the inhibition of cell death via the deletion of TRIF and PKR or the Z-VAD treatment (Fig. 7b, c, e, f). PKR and TRIF have each been shown to play an important role in the process of antiviral immunity. PKR inhibits viral protein translation and enhances viral RNA recognition by forming SG [21, 31], while TRIF is required for TLR3-dependent IFN-I production [15]. We herein demonstrated their importance from the perspective of the induction of cell death as well as the resulting inhibitory effects on viral replication. We concluded that viral dsRNA induced distinct signals in infected cells to initiate the antiviral program mediated by antiviral protein production and the rapid death program mediated by TRIF and PKR. Defects in PKR or TRIF signaling allow the virus to make greater use of host cells for proliferation and lead to more severe infections.

In addition, our research developed the application of microinjections, which allows investigations on the stimulatory effects of nucleic acids, proteins, and their mixtures in single cells. This method is advantageous for examining the immunostimulatory features of in vitro transcribed nucleic acid products and the function of native undenatured proteins.

## Materials and methods

### Cells

Wild-type HeLa cells (#CCL-2.2, ATCC) and derivatives were cultured with high glucose DMEM (Nacalai Tesque) containing 5% fetal bovine serum (FBS, Gibco) and 1% penicillin–streptomycin (P/S Nacalai Tesque) (c-DMEM) at 37 °C in a 5% CO_2_ incubator. Cell lines were regularly treated with plasmocin (InvivoGen) to avoid mycoplasma contamination.

HeLa cells with RIG-I KO, MDA5 KO, IPS-1 KO, IRF-3 KO, IFNAR1 KO, PKR KO, TRIF KO, and TRIF PKR DKO were generated by the CRISPR-cas9 method using the backbone plasmid pSpCas9(BB)-2A-GFP (PX458), a gift from Dr. Feng Zhang (Addgene plasmid #48138), sorted by GFP using SH800S Cell Sorter (SONY), and subjected to single-cell selection. The sgRNA sequences for each target were:

RIG-I sgRNA: forward 5’-GGATAAGATGGAAACTTCTGACA-3’,

RIG-I sgRNA: reverse 5’-GGCCTGAAGATCCTCCAAGT-3’;

MDA5 sgRNA: forward 5’-TGGTTGGACTCGGGAATTCG-3’,

MDA5 sgRNA: reverse 5’-CGAATTCCCGAGTCCAACCA-3’;

IPS-1 sgRNA: forward 5’-CCTGGTGCAGTGCCTTCTA-3’,

IPS-1 sgRNA: reverse 5’-GTGACTACCAGCACCCCTGT-3’;

IRF-3 sgRNA: forward 5’-TCCACCATTGGTGTCCGGAG-3’,

IRF-3 sgRNA: reverse 5’-CTCCGGACACCAATGGTGGA-3’;

IFNAR1 sgRNA: forward 5’-CACCAAGCAGCACTACTTACGTCA-3’,

IFNAR1 sgRNA: reverse 5’-TGACGTAAGTAGTGCTGCTTCAAA-3’;

PKR sgRNA: forward 5’-CAGTGTGCATCGGGGGTGCAGTTT-3’,

PKR sgRNA: reverse 5’-TGCACCCCCGATGCACACTGCGGTG-3’;

TRIF sgRNA: forward 5’-ATGAGGCCCGAAACCGGTGTGGG-3’,

TRIF sgRNA reverse: 5’-CCCACACCGGTTTCGGGCCTCAT-3’.

GFP-IRF-3 and GFP-Δ1-58IRF-3 HeLa cells were generated by transfecting respective expression vectors into IRF-3 KO HeLa cells. Forty-eight hours after transfection, cells were collected for GFP fluorescence by cell sorting as described above and selected by G418 (Nacalai Tesque) resistance in 10-cm dishes for two weeks, followed by single-cell selection. GyrB-PKR and GyrB-PKR K296H HeLa cells were generated by co-transfection with respective expression vectors and a selection marker (pIRES puro2, Clontech) into PKR KO HeLa cells. Forty-eight hours after transfection, cells were selected for puromycin (InvivoGen) resistance for 2 weeks, followed by single-cell selection. GyrB-PKR FK-IPS-1 HeLa cells were generated by transfecting the expression vector for FK-IPS-1 into GyrB-PKR HeLa cells, followed by G418 selection for 2 weeks and single-cell selection.

### Plasmid constructs

GFP-IRF-3 and GFP-Δ1-58IRF-3 constructs were generated by inserting the coding sequence of IRF-3 (1-427) and (59-427), respectively, into the pAcGFP1-C1 expression vector (Clontech). The expression vectors pC939 GyrB-PKR and pC940 GyrB-PKR K296H were kindly provided by Dr. Tom Dever [40]. The expression vectors for the FK-IPS-1 construct were previously described [41]. The cFLIP expression vector was constructed by inserting full-length cFLIP cDNA (amplified by primers, forward: 5’-ATGTCTGCTGAAGTCATCCA-3’ and reverse: 5’-TTATGTGTAGGAGAGGATAAG-3’) into the pEF-BOS( + ) vector [52].

### Microinjection and live cell imaging

(1) Cells subjected to microinjections were seeded at 1 × 10^5^ on 35-mm culture dishes (Greiner Bio-One) with a grid-imprinted cover glass (Matsunami Glass #CS01885). Cells were cultivated for 48 h.

(2) Cover glasses were transferred using forceps into a μl-Dish 35 mm high plate (ibidi) with 1 ml of 37 °C pre-warmed PBS. Colonies of 8–32 cells were selected under a confocal microscope (Leica TCS SP8). Images taken at this time point were annotated as (0 h).

(3) Cells were manually microinjected using Leica MICROSYSTEMS with a self-made glass needle (radius <0.5 μm) at room temperature (RT). Cover glasses carrying cells were placed on the injection glass plate and covered with 400 µl of c-DMEM. Injection substrates were centrifuged at 12,000×*g* at 4 °C for 10 min and 2 μl of the substrate was then collected and loaded into the injection glass needle by micro-tips (Eppendorf). The glass needle connected to the injection pump (FemtoJet, Eppendorf) was initially flushed with c-DMEM to test the mobility of the substrate and remove the remaining air bubbles. The appropriate injecting pressure for the presenting substrate was optimized using neighborhood cells. A pressure that enables a small stream of the substrate to flow into the cell cytoplasm without stretching the cell body was selected and used for subsequent injections. Selected colonies were injected into the cytoplasm in one shot. After the injection, the cover glass was transferred back to the cell culture dish for further cultivation.

(4) Cells were subjected to confocal imaging at the indicated time points after the injection using grid numbers in the coverslip as a reference.

The workflow of microinjections is depicted in Supplementary Fig. S1a.

All injection experiments were independently performed at least three times. In each repeat and for each substrate, 3–6 colonies were injected. PBS was easily injected at a pressure of ~80 hundred pascals (hPa), whereas concentrated poly I:C and proteins required higher injecting pressures of ~150–180 hPa. An excessive injecting pressure caused cell bursting, as described in Supplementary Fig. S2a. The average injection volume was approximately one-tenth of the total cell volume. The radius of a HeLa cell is ~12.5 µm; therefore, considering the hemispherical shape of the attached cell, the average volume of the injected substrate was estimated to be (*π*×12.5^2^) × 1/2 × 1/10) ≈ 24.5 *fl*.

### Preparation of injection substrates

The short 5’-ppp-RNA, GG25 [35] was produced using the AmpliScribe™ T7-Flash™ Transcription Kit (Epicentre) according to the manufacturer’s protocol with the dsDNA template generated by annealing two synthetic DNA: 5’-TAATACGACTCACTATA-3’ and: 5’-CACTTTCACTTCTCCCTTTCAGTTTCCTATAGTGAGTCGTATTA-3’. After incubation at 37 °C for 4 h, the reaction was digested with DNase I, and RNA was purified by phenol/chloroform extraction, Mini Quick Spin Columns (Roche), and ethanol precipitation. Poly I:C was from GE Healthcare. Recombinant RIG-I was produced in High Five cells using recombinant baculovirus and purified [53]. Recombinant ∆TM-IPS-1 was produced from *Escherichia coli* expressing ∆TM-IPS-1 and purified [54]. A mixture of RIG-I and GG25 was prepared by mixing (1 µg/µl each) followed by incubation at 37 °C for 30 min just prior to the injection. Rb-dsRNA, the genome of rice *Endornavirus*, was extracted from rice bran as previously described [38].

### Chemical reagents

Dimethyl sulfoxide (DMSO), Cycloheximide (CHX), and chloroquine were purchased from Nacalai Tesque, coumermycin A1 from Promega, human TNF-α from PeproTech, AP20187 from ARIAD Pharm, Z-VAD from R&D Systems, NH_4_Cl from SI Science, and human IFN-β from Sigma.

### Immunoblotting

Cells were harvested in cold PBS and lysed with RIPA buffer (150 mM NaCl, 50 mM Tris-HCl pH 7.5, 1% NP40, 0.5% DOC, and 0.5% SDS) containing protein inhibitor cocktail (1:1000) on ice. They were then incubated at 4 °C for 30 min followed by centrifugation (16,000 × *g*, 4 °C, 10 min). The supernatant was collected and subjected to SDS-PAGE or native-PAGE (IRF-3 dimerization). Proteins were transferred to an Immobilon-P PVDF membrane (Millipore). The membrane was blocked in TBS-T containing 5% skim milk (blocking buffer) at RT for 30 min. Primary antibodies were diluted in blocking buffer at 1:1000 or 1:500 (cFLIP) and incubated at 4 °C overnight. Horseradish peroxidase (HRP)-conjugated secondary antibodies were diluted at 1:3000 in blocking buffer or TBS-T (cFLIP) and incubated at RT for 1 h. Protein signals were visualized using Chemi-Lumi One Super (Nacalai Tesque) and the LAS-4000 instrument (Fujifilm).

Antibodies were from: Cosmo Bio, anti-IRF-3 mouse mAb (CBX-CBX00167); Enzo Life Sciences, anti-cFLIP mouse mAb (#ALX-8040961-0100); Santa Cruz Biotechnology, anti-PKR mouse mAb (#sc-6282), anti-GAPDH mouse mAb (#sc-32233); Cell Signaling Technology, anti-TRIF rabbit mAb (#4596S), anti-PARP rabbit mAb (#9542S), anti-caspase 8 mouse mAb (#9745S), anti-caspase 9 rabbit mAb (#9502S), HRP-linked anti-mouse IgG (#7076S), and HRP-linked anti-rabbit IgG (#7074S).

### Transfection and siRNA

Cells were seeded at 50% confluency 24 h prior to transfection. Cells were washed with PBS. Expression vector plasmids, GG25, and poly I:C were transfected with Lipofectamine 2000 (Invitrogen) in Opti-MEM (Gibco). siRNA was transfected with Lipofectamine RNAiMAX (Invitrogen) in Opti-MEM. siRNA for cFLIP and control RNA were from Applied Biosystems.

### Virus infection and titration

Cells were seeded on 12-well plates (2 × 10^5^ cells/well) 24 h prior to infection. Cells were washed with PBS and infected with SeV (3.2 × 10^2^ HAu/ml) and SINV (MOI = 1), respectively. One hour after infection, the virus was removed and replaced by 500 μl of fresh c-DMEM. Supernatants were collected at the indicated time points for viral titration. A hemagglutination assay was performed for SeV using chicken erythrocytes (Japan Bio Science Laboratory). The culture supernatant was serially diluted two-fold in a round-bottomed 96-well microplate (50 µl) and mixed with 50 µl of 0.5% erythrocyte suspension. The plate was incubated at RT for 1 h and aggregation was assessed. The titer of SINV was measured by a plaque assay using Vero cells. In total, 1 × 10^5^ Vero cells were seeded in a 24-well plate for 24 h and infected with the serially diluted cell culture supernatant. One hour after infection, medium was replaced with 1 ml of c-DMEM containing 1.5% Avicel (Sigma). Forty-eight hours after SINV infection, c-DMEM containing Avicel was removed, cells were gently washed by PBS, fixed with 4% PFA, and stained by crystal violet (Nacalai Tesque) at RT for 20 min. Plates were washed, dried, and subjected to plaque counting.

### RNA fluorescence in situ hybridization (FISH)

Coverslips used for microinjection were transferred to a 24-well plate by forceps, gently washed with 500 μl of PBS, and fixed in 300 μl of 4% PFA at RT for 15 min. After removing PFA, coverslips were washed with 500 μl of PBS twice. Cells were permeabilized with 300 μl of 0.1% Triton X-100 (PBS) at RT for 15 min, followed by washing with PBS. FISH was performed using a kit (Affymetrix) according to the manufacturer’s protocol. In brief, cells were initially incubated with the human *IFNB* probe (1:100) in probe set buffer at 40 °C for 3 h, followed by incubation with a pre-amplifier, amplifier, and label probe (1:25) in the respective buffers. Cells were then washed three times with FISH washing buffer between each step and stained with DAPI (1:1000) in PBS for 10 min. Coverslips were mounted on a glass slide and imaged with a Leica TCS SP8 confocal microscope.

### Immunostaining

Cell fixation and permeabilization were conducted as described in the FISH method.

Cells were blocked in PBS-T containing 1% BSA and 5% glycerol at 4 °C for 30 min. The primary antibody was added (1:500) to 1% BSA-blocking buffer and incubated at 4 °C overnight. Cells were washed three times with PBS-T for 10 min. The secondary antibody (1:1000) in 1% BSA-blocking buffer was added and incubated at 4 °C for 1 h, followed by three washes with PBS-T. Nuclei were stained with DAPI (1:1000) in PBS-T at RT for 10 min and cells were washed twice with PBS-T. Coverslips were mounted on a glass slide and imaged with a Leica TCS SP8 confocal microscope. The following antibodies were used: anti-IRF-3 rabbit pAb [37]. Santa Cruz Biotechnology, anti-G3BP1 mouse mAb (#sc-365338), anti-TIAR goat pAb (#sc-1749); Life Technologies, Alexa Fluor 488 donkey anti-rabbit, Alexa Fluor 594 donkey anti-mouse, and Alexa Fluor 647 donkey anti-goat.

### Cell survival quantification

% Survival of injected cells was calculated by the direct counting of dead cells using the following formula:

$${{{\mathrm{Cell}}}}\;{{{\mathrm{survival}}}} = \left( {1 - \frac{b}{a}} \right) \times 100\%$$

a = the total number of injected cells (deduct mechanical cell death)

b = the number of currently surviving cells.

Cells subjected to Amido black staining were gently washed with PBS and fixed in 4% PFA for 15 min. Amido black was added at RT for 30 min and then washed. Plates were dried overnight. Regarding quantification, cells were washed with 0.3 M CH_3_COONa (pH 5.6) to reduce the background, and Amido black was extracted by an incubation (300 μl/well) with 50 mM NaOH. The absorbance of Amido black was quantified by a microplate reader (Bio-Rad) at 630 and 405 nm. OD_630_−OD_405_ was calculated as the Amido black intensity (ABI) and used in the cell survival analysis.

### RNA extraction and real-time qPCR

Total RNA was extracted from cells with TRIzol reagent according to the lab protocol and reverse transcribed using the high-capacity cDNA reverse transcription kit (Applied Biosystems). cDNA was amplified with the Fast SYBR green master mix (Applied Biosystems) on a Step One Plus real-time PCR system (Applied Biosystems).

SYBR green primers used in the present study were:

*h-GAPDH* forward: 5’-CTGCACCACCAACTGCTTAG-3’

*h-GAPDH* reverse: 5’-GTCTTCTGGGTGGCAGTGAT-3’

*h-IFNB* forward: 5’-AGTCTCATTCCAGCCAGTGC-3’

*h-IFNB* reverse: 5’-AGCTGCAGCAGTTCCAGAAG-3’

*h-cFLIP* forward 5’-CTGGTTGCCCCAGATCAACT-3’

*h-cFLIP* reverse 5’-CCCAGGGAAGTGAAGGTGTC-3’.

### Statistical analysis

Statistical analyses were performed using GraphPad Prism, data represent means ± SEM, *N* = 3, and significance is shown as *P < 0.05, **P < 0.01, ***P < 0.001, ****P < 0.0001, and ns: not significant. Microscopy and immunofluorescence images are representative of at least three independent experiments. All experiments shown were replicated in the laboratory more than six times.

## Supplementary information

Supplemental figure legends

Figure S1

Figure S2

Figure S3

Figure S4

Figure S5

Figure S6

Figure S7

Original Western Blot

Reproductivity checklist

## Data Availability

All data supporting the results reported in the article are available from the corresponding author upon reasonable request.
