# Correction: PKR and TLR3 trigger distinct signals that coordinate the induction of antiviral apoptosis

**DOI:** 10.1038/s41419-022-05227-4

**Published:** 2022-09-19

**Authors:** Wenjie Zuo, Mai Wakimoto, Noriyasu Kozaiwa, Yutaro Shirasaka, Seong-Wook Oh, Shiori Fujiwara, Hitoshi Miyachi, Amane Kogure, Hiroki Kato, Takashi Fujita

**Affiliations:** 1 Division of Integrated Life Science, Graduate School of Biostudies, Kyoto University, Sakyo-ku, Kyoto, 606-8507 Japan; 2 Laboratory of Regulatory Information, Institute for Frontier Life and Medical Science, Kyoto University, Sakyo-ku, Kyoto, 606-8507 Japan; 3 Institute for Virus Research, Kyoto University, Sakyo-ku, Kyoto, 606-8507 Japan; 4 Institute for Cardiovascular Immunology, University Hospital Bonn, Bonn, 53127 Germany

**Keywords:** Cell death and immune response, Signal transduction, Pattern recognition receptors, Oligo delivery, Imaging the immune system

Correction to: *Cell Death and Disease* 10.1038/s41419-022-05101-3, published online 15 August 2022

The original version of this article contained an error in the text and the catalog number of the antibody was not correct. The authors apologize for the errors. The original article has been corrected.

