# Supplementary material for: PKR and TLR3 trigger distinct signals that coordinate the induction of antiviral apoptosis
Source: Cell Death Dis. 2022 Aug 15;13(8):707. doi: 10.1038/s41419-022-05101-3 (PMC9378677; doi:10.1038/s41419-022-05101-3)
Supplement: Supplementary file 1 — Supplemental figure legends [file 41419_2022_5101_MOESM1_ESM.pdf]

Supplemental figure legends

Figure S1. Microinjection workflow and *IFNB* mRNA induction

a: Microinjections were conducted as described in each step (See Methods for details).

b: GFP-IRF-3 HeLa cells were injected with GG25 (1 µg/µl) alone. Cells were fixed after 3 h and observed for IRF-3 (green) or the expression of *IFNB* mRNA (FISH, red). Cells encircled in red are injected cells. Cells encircled in green show nuclear IRF-3. Arrows indicate cells positive for *IFNB* mRNA.

c: GFP-IRF-3 HeLa cells injected with GG25 alone or RIG-I+GG25 were analyzed as in b and quantified for the localization of IRF-3 and expression of *IFNB* mRNA.

Figure S2. Morphological differences between RNA-induced cell death and that caused by mechanical stress

a: GFP-IRF-3 HeLa cells were injected with PBS at a low (100 hPa) or high (200 hPa) influx pressure and observed live for GFP and cell death. Injected cells are encircled in red. Cells that died due to a high influx pressure are encircled in white.

b: GFP-IRF-3 HeLa cells were injected with poly I:C (1 µg/µl) and observed live for different types of cell death. Injected cells are encircled in the red. RNA-induced dead cells are shown by red arrows. Cells that died due to mechanical stress are indicated by white arrows.

Figure S3. Apoptosis induced by GG25 and poly I:C transfection

a: GFP-IRF-3 HeLa cells were mock transfected (lipofectamine only) or transfected with poly I:C (0.5 µg/ml, 24 h) or GG25 (4 µg/ml, 24 h) with lipofectamine and observed for cell death. Scale bar = 50 µm

b: HeLa cells were pre-treated with culture medium with or without IFN-β (1000 U/ml) for 12 h, transfected with GG25 (4 µg/ml), and examined for cell survival at the indicated time points.

c: HeLa cells were pre-treated with culture medium with or without IFN-β (1000 U/ml) for 12 h, transfected with poly I:C (0.5 µg/ml), and examined for cell survival at the indicated time points. Data in (b) (c) are represented as the means ± SEM of two independent experiments.

Figure S4. Diverse mechanisms underlying cell death induced by GG25 and poly I:C.

a: The expression of the GFP-IRF-3 and GFP-Δ1-58IRF-3 fusion protein in stably expressing HeLa cells was examined by immunoblotting; the dimerization of GFP-IRF-3 and GFP-Δ1-58IRF-3 was confirmed by native-PAGE and immunoblotting upon SeV infection (12 h). The relative expression of the *IFNB* gene in GFP-IRF-3 and GFP-Δ1-58IRF-3-stably-expressing HeLa cells upon GG25 transfection (4 µg/ml, 12 h) was examined by RT-qPCR. The means + SEM of three independent experiments are shown; data were analyzed by a two-way ANOVA followed by Tukey's multiple comparisons test; ****$P < 0.0001$; ns, not significant.

b: The levels of cleaved PARP in GFP-IRF-3, GFP-Δ1-58IRF-3, and GFP-IRF-3 IFNAR1 KO HeLa cells upon the transfection of GG25 (4 μg/ml, 24 h) were examined by immunoblotting.
c: GFP-Δ1-58IRF-3 and GFP-IRF-3 IFNAR1 KO HeLa cells were injected with poly I:C (1 μg/μl) and observed live for the localization of IRF-3 and cell death at the indicated times after the injection. % Cell death was calculated and shown in red.
d. Wild-type and the indicated KO HeLa cells were transfected with poly I:C (0.5 μg/ml, 24 h) and observed for cell death.
e. GFP-IRF-3 HeLa cells were injected with rb-dsRNA (1 μg/μl) and observed live for the localization of IRF-3 and cell death at the indicated times after the injection. % Cell death was calculated and shown in red.
Scale bar = 25 μm in (c) (e) and 100 μm in (d).

Figure S5. Construction of the GyrB-PKR and GyrB-PKR FK-IPS-1 activation system.
a: The expression of the GyrB-PKR and GyrB-PKR K296H fusion protein in stably expressing HeLa cells was examined by immunoblotting.
b: GyrB-PKR HeLa cells were mock treated (DMSO) or treated with coumermycin A1 at the indicated concentration for 12 h and then examined for cell survival. The means + SEM of three independent experiments are shown; data were analyzed by a one-way ANOVA followed by Dunnett’s multiple comparisons test; ns, not significant.
c: Schematic representation of the IPS-1 and FK-IPS-1 fusion protein and activation mechanism.
d: GyrB-PKR FK-IPS-1 HeLa cells were mock treated (DMSO) or treated with AP20187 and coumermycin A1 (10 nM each) simultaneously for 1 h, and cells were then fixed and stained for IRF-3 and G3BP1 with the respective antibodies for microscopy. Scale bar = 50 μm.

Figure S6. Cell death induced by cytoplasmic poly I:C requires PKR and TRIF
a: The expression of PKR and TRIF in PKR KO, TRIF KO, and PKR TRIF DKO HeLa cells was examined by immunoblotting.
b: Wild-type and the indicated KO HeLa cells were left untreated (Mock) or treated with poly I:C (5 μg/ml, 24 h) or transfected with poly I:C (0.5 μg/ml, 24 h), and were then examined for cell survival. The means + SEM of three independent experiments are shown; data were analyzed by a two-way ANOVA followed by Tukey’s multiple comparisons test; ***$P < 0.001$, ****$P < 0.0001$; ns, not significant.
c: GFP-IRF-3, PKR KO, TRIF KO, and PKR TRIF DKO HeLa cells were injected with poly I:C (1 μg/μl) for 3 h, and then fixed and stained for IRF-3 (except GFP-IRF-3 HeLa cells) and G3BP1 with the respective antibodies for microscopy. Scale bar = 25 μm.

Figure S7. PKR activation promoted the TNF-α-induced activation of extrinsic apoptosis.

a: Wild-type and the indicated HeLa cells were transfected with poly I:C (0.5 µg/ml) and examined for cell survival at the indicated time points.

b: GyrB-PKR HeLa cells were mock treated (DMSO) or treated with the indicated stimulant for 12 h and examined for cell survival. The means + SEM of three independent experiments are shown; data were analyzed by a one-way ANOVA followed by Dunnett's multiple comparisons test; ****$P < 0.0001$; ns, not significant.
