# Supplementary figures and images for: PKR and TLR3 trigger distinct signals that coordinate the induction of antiviral apoptosis
Source: Cell Death Dis. 2022 Aug 15;13(8):707. doi: 10.1038/s41419-022-05101-3 (PMC9378677; doi:10.1038/s41419-022-05101-3)

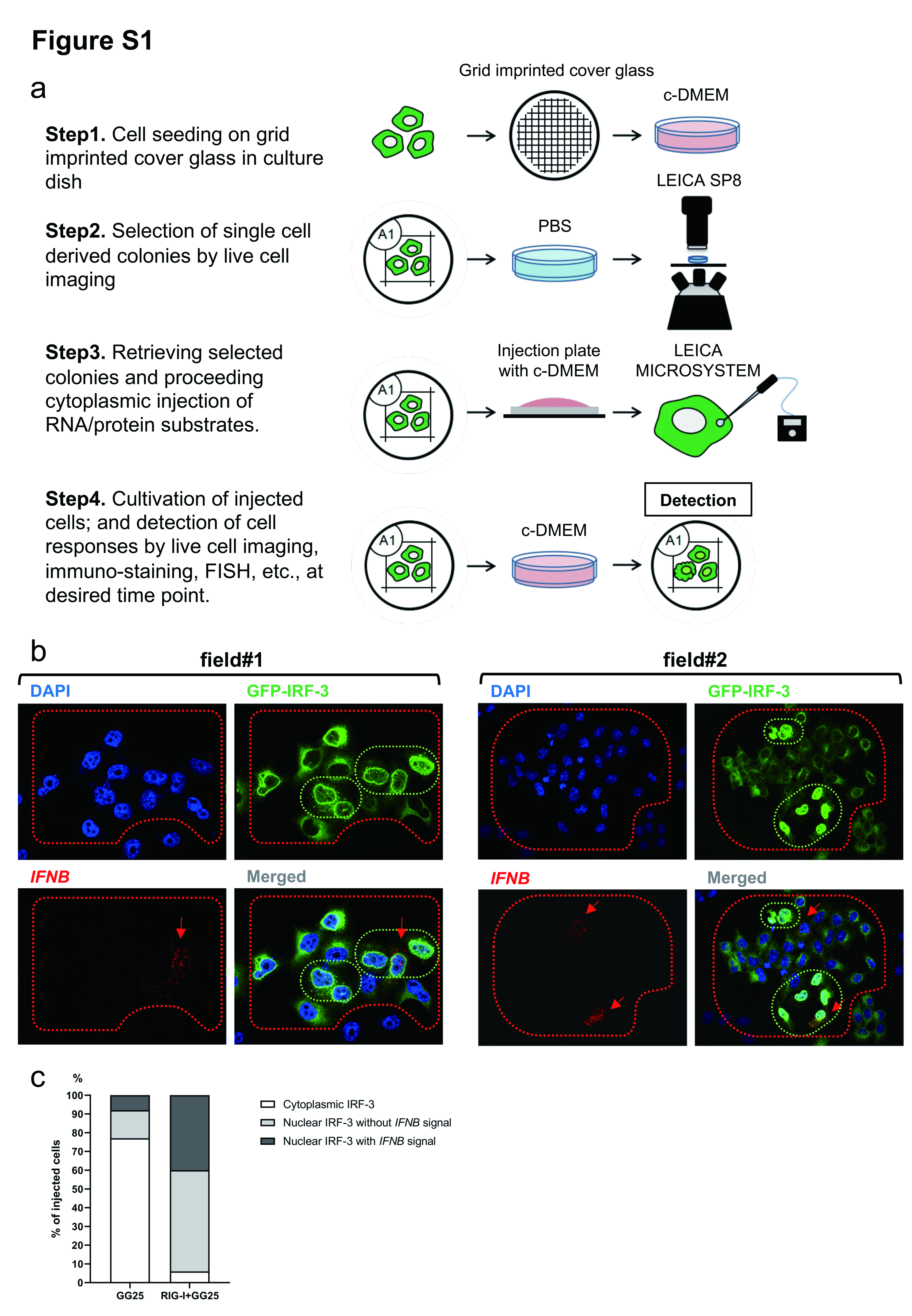

Supplement: Supplementary file 2 — Figure S1 [file 41419_2022_5101_MOESM2_ESM.jpg]

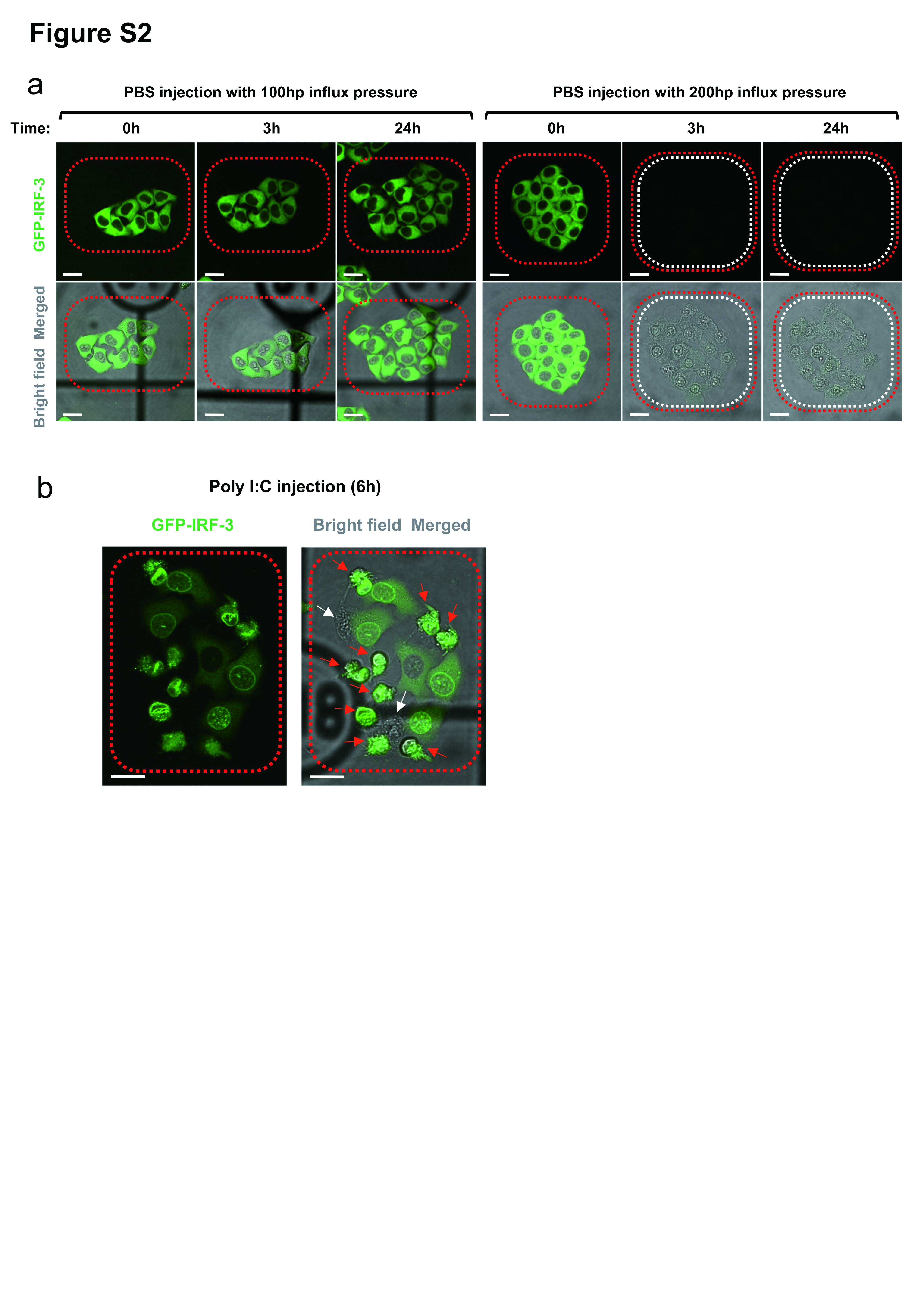

Supplement: Supplementary file 3 — Figure S2 [file 41419_2022_5101_MOESM3_ESM.jpg]

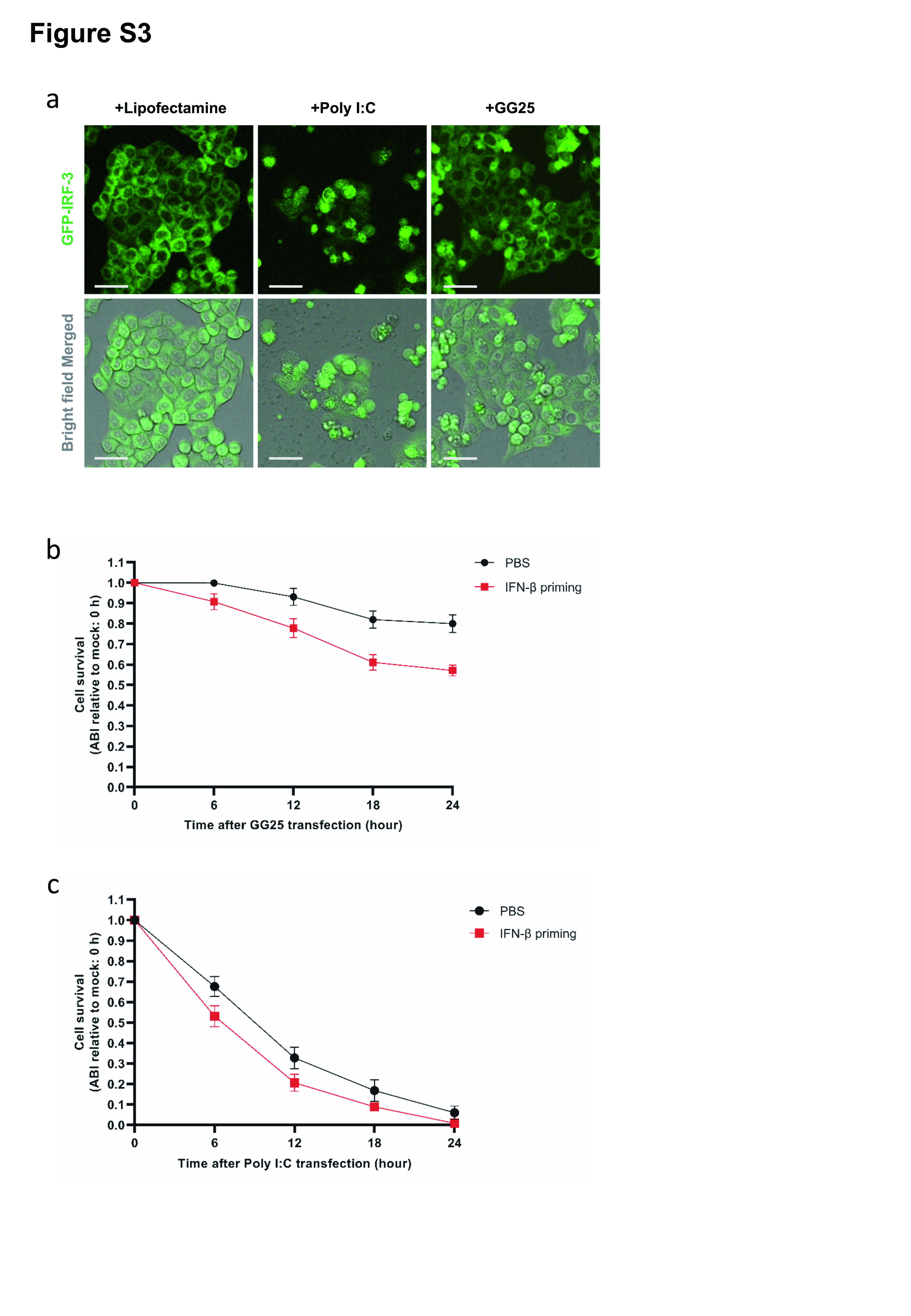

Supplement: Supplementary file 4 — Figure S3 [file 41419_2022_5101_MOESM4_ESM.jpg]

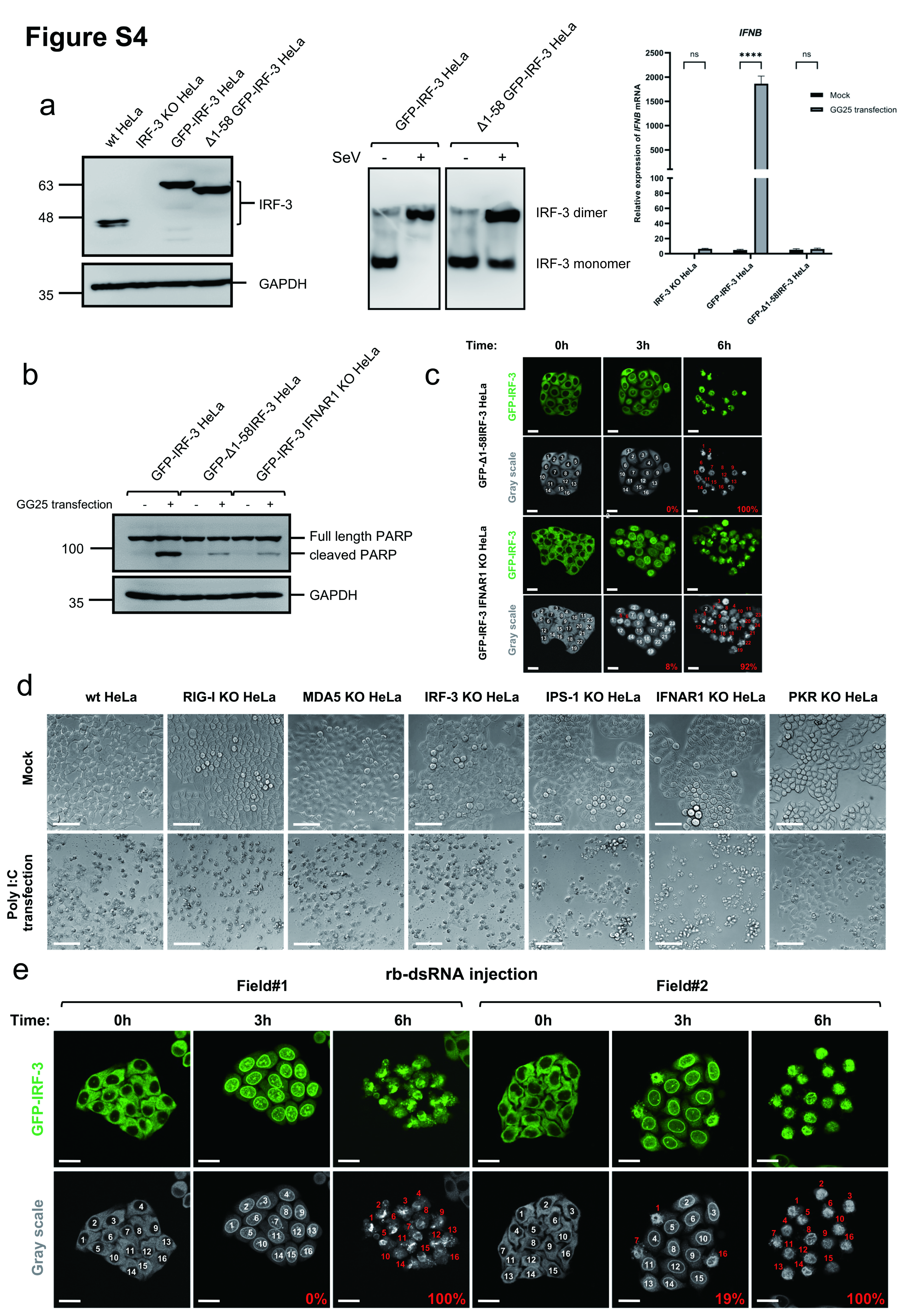

Supplement: Supplementary file 5 — Figure S4 [file 41419_2022_5101_MOESM5_ESM.jpg]

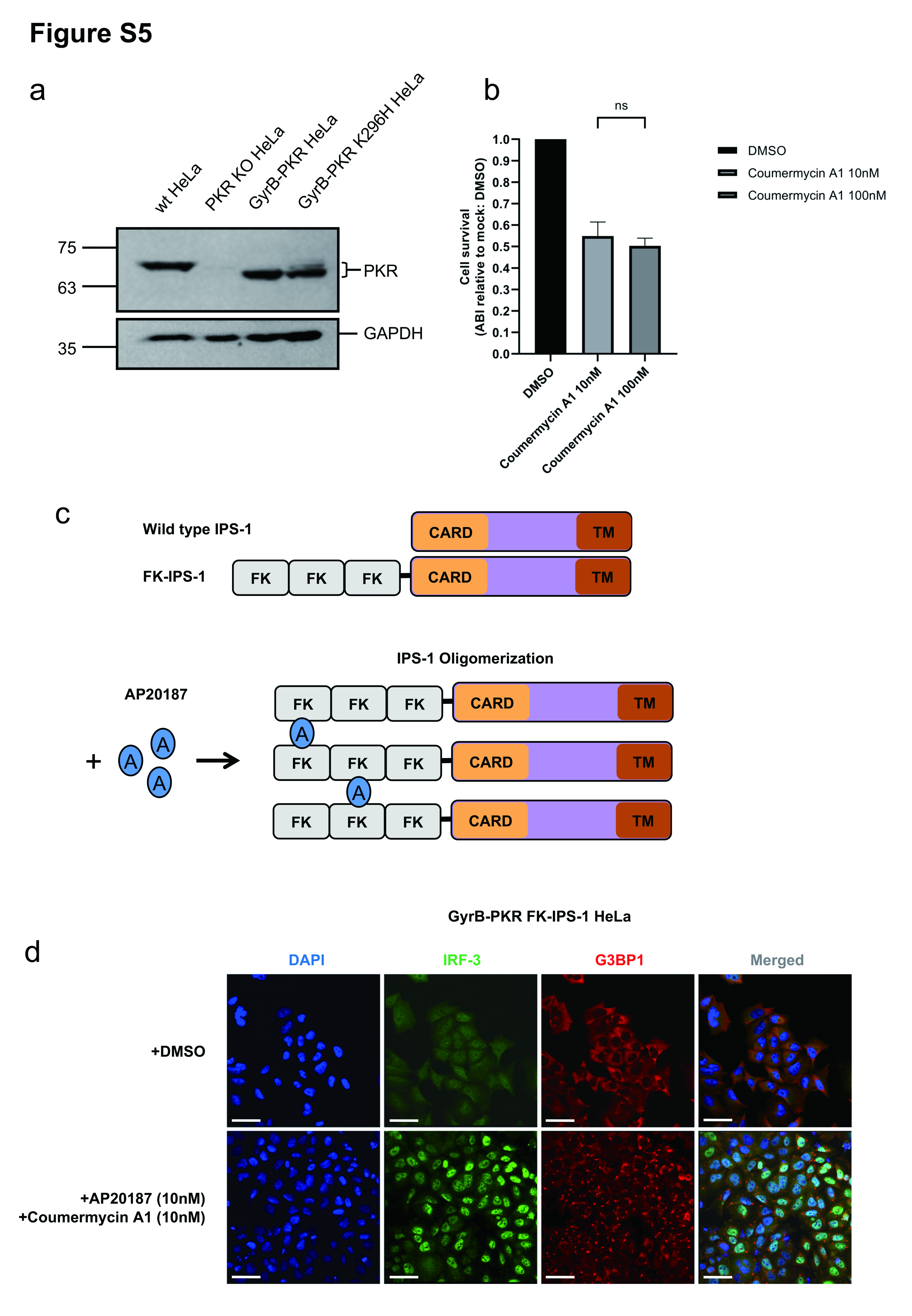

Supplement: Supplementary file 6 — Figure S5 [file 41419_2022_5101_MOESM6_ESM.jpg]

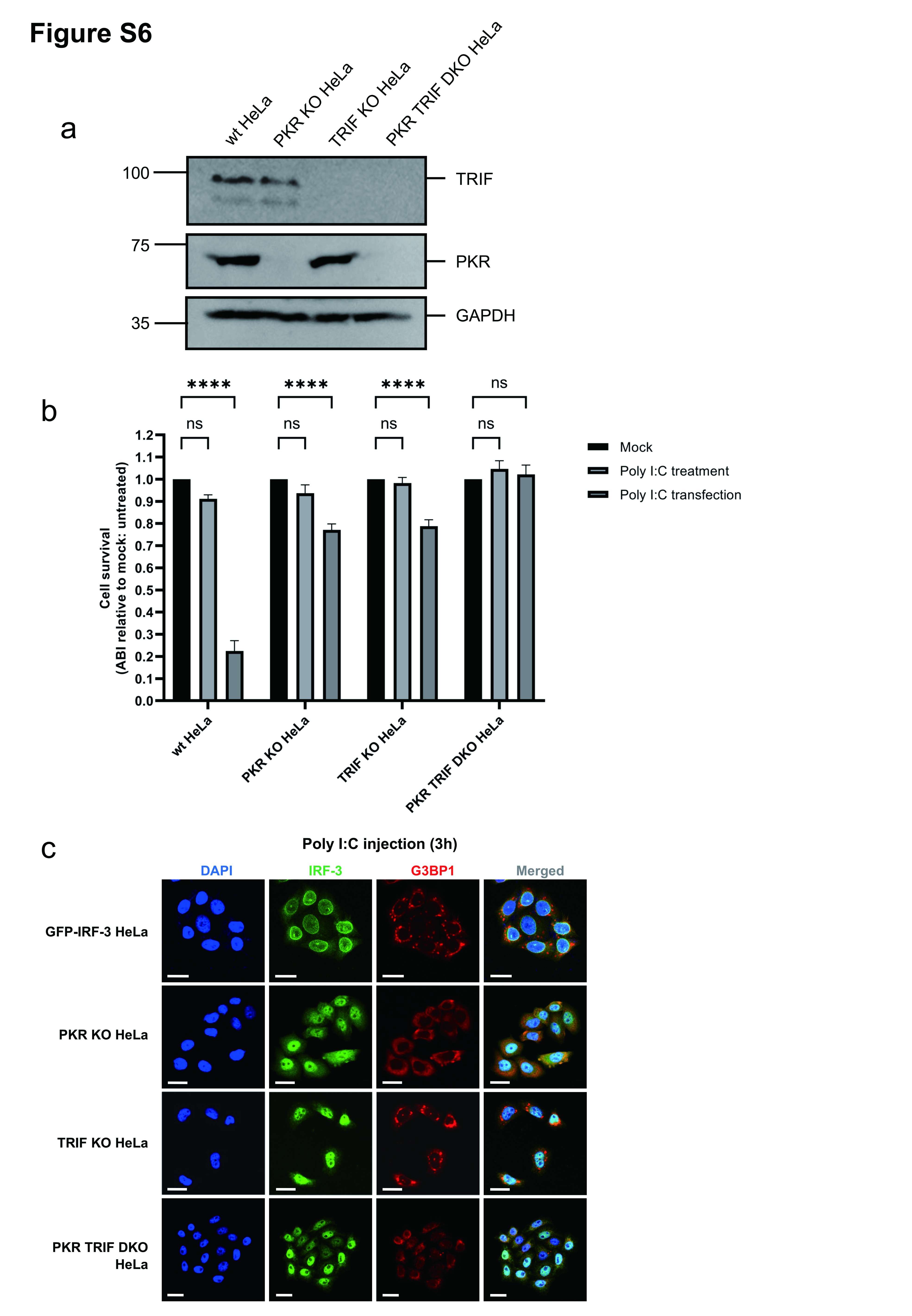

Supplement: Supplementary file 7 — Figure S6 [file 41419_2022_5101_MOESM7_ESM.jpg]

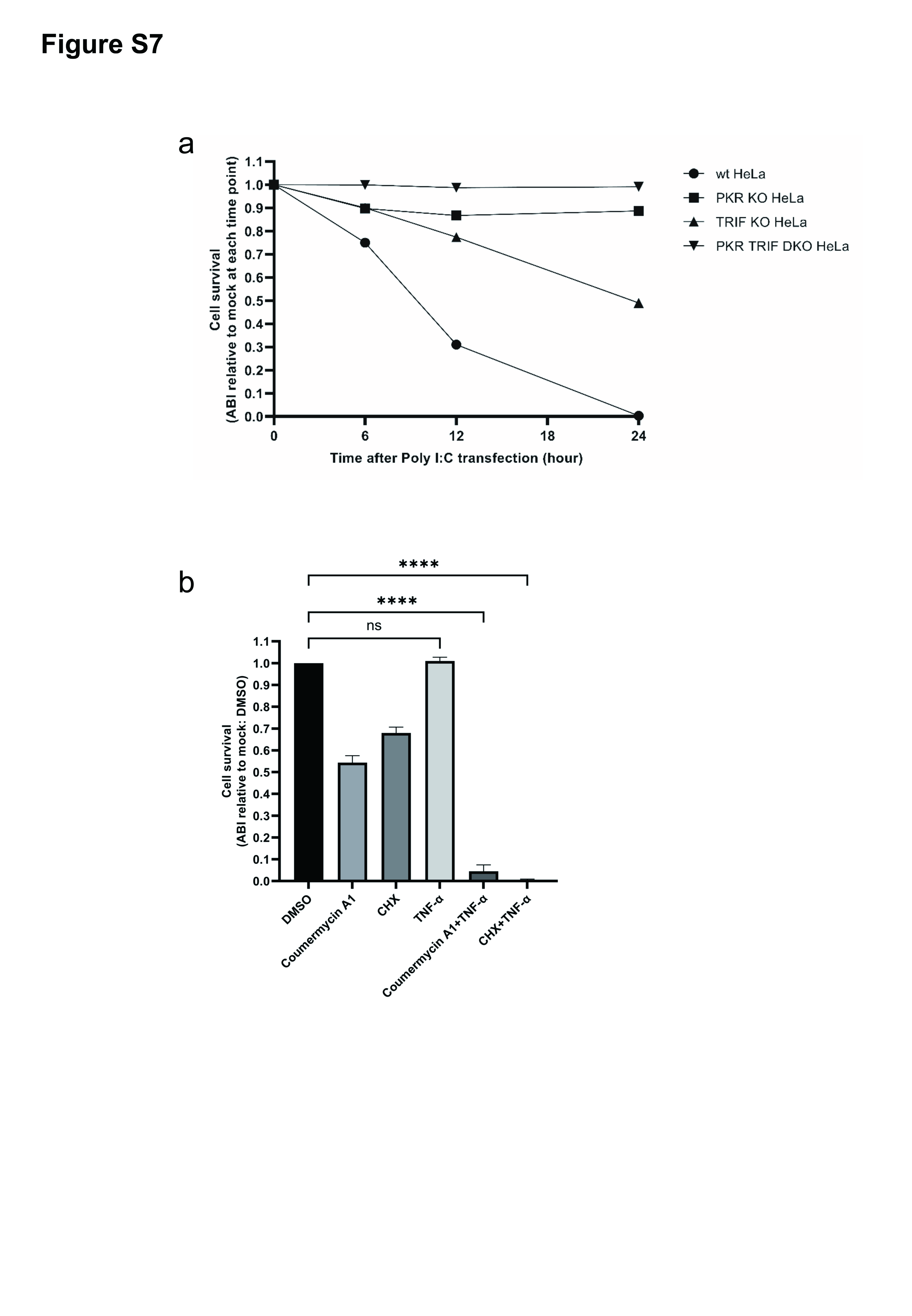

Supplement: Supplementary file 8 — Figure S7 [file 41419_2022_5101_MOESM8_ESM.jpg]
