# Supplementary material for: PKR and TLR3 trigger distinct signals that coordinate the induction of antiviral apoptosis
Source: Cell Death Dis. 2022 Aug 15;13(8):707. doi: 10.1038/s41419-022-05101-3 (PMC9378677; doi:10.1038/s41419-022-05101-3)

Original western blots 1

Figure 6a

cFLIP

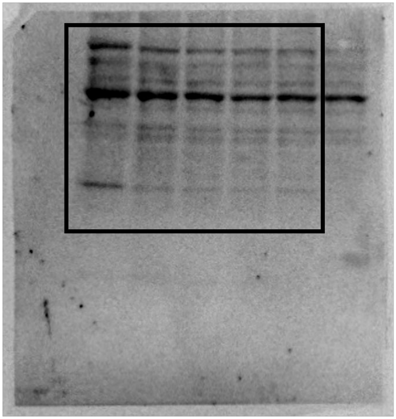

GAPDH

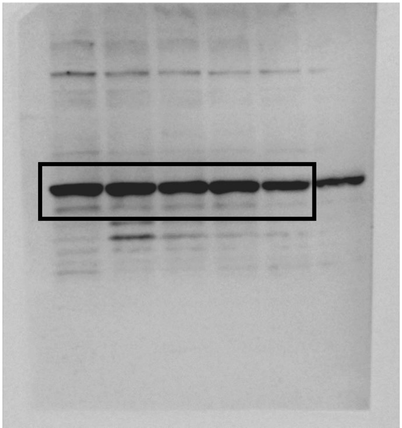

Figure 6b

cFLIP

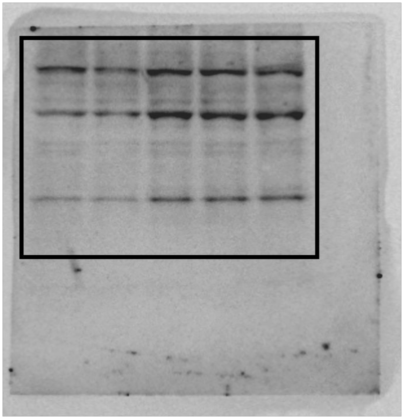

GAPDH

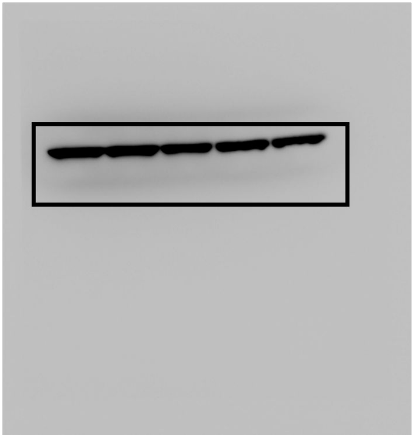

Original western blots 2

Figure 6d

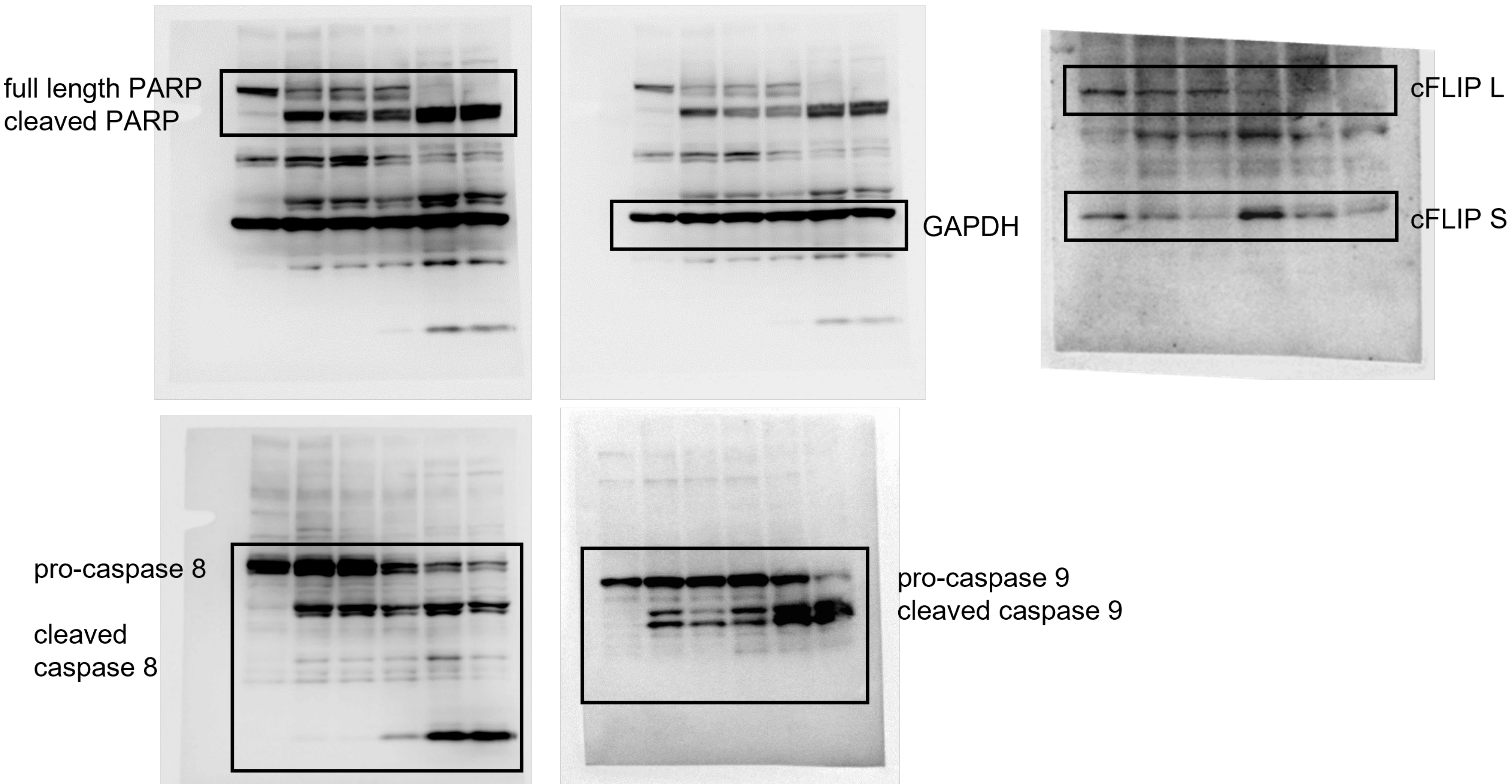

Original western blots 3

Figure 6g

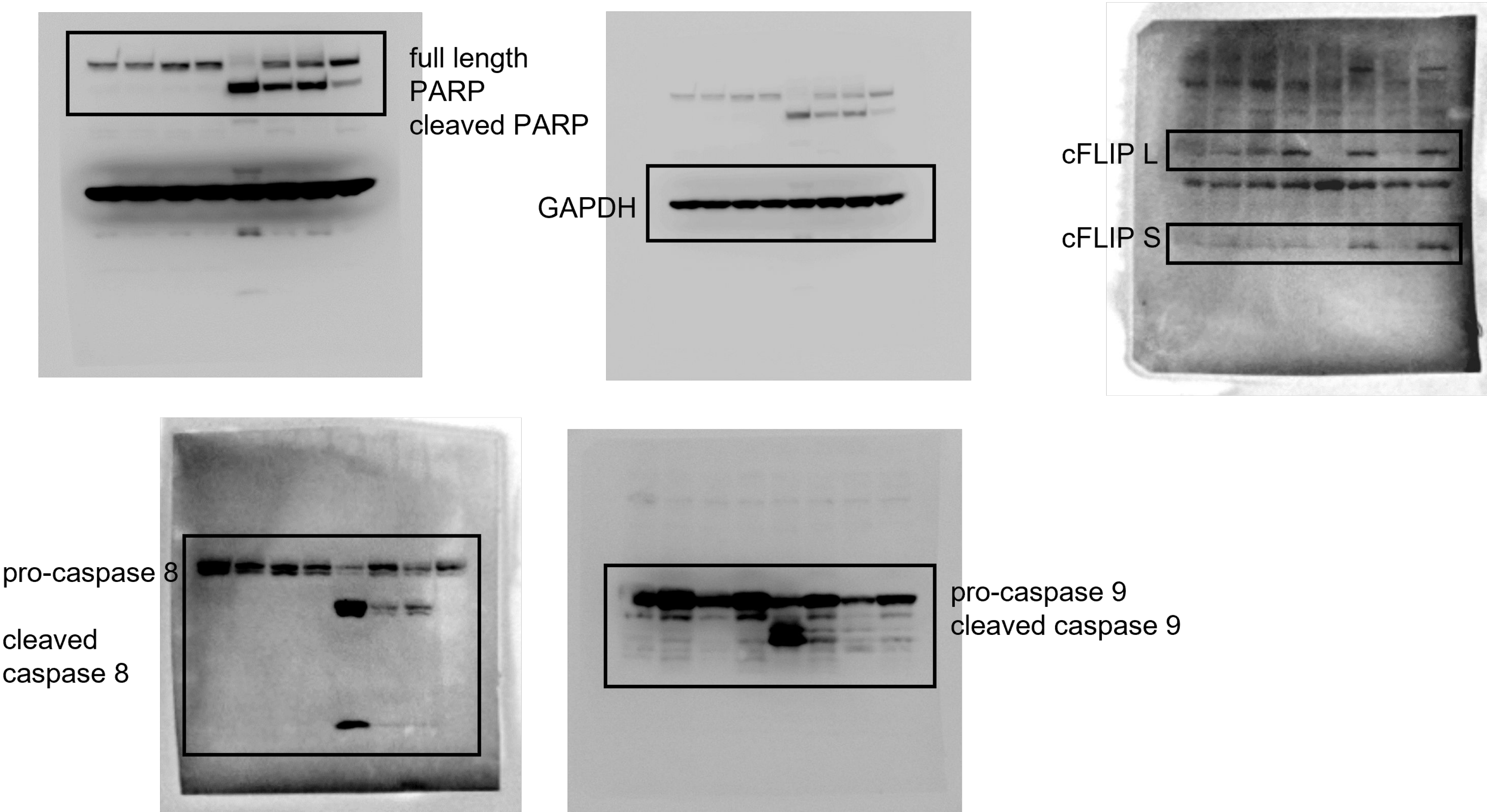

Supplement: Supplementary file 9 — Original Western Blot [file 41419_2022_5101_MOESM9_ESM.pdf]
